# STAT3 pathway in cancers: Past, present, and future

**DOI:** 10.1002/mco2.124

**Published:** 2022-03-23

**Authors:** Han‐Qi Wang, Qi‐Wen Man, Fang‐Yi Huo, Xin Gao, Hao Lin, Su‐Ran Li, Jing Wang, Fu‐Chuan Su, Lulu Cai,, Yi Shi, Bing Liu,, Lin‐Lin Bu

**Affiliations:** ^1^ The State Key Laboratory Breeding Base of Basic Science of Stomatology (Hubei‐MOST) & Key Laboratory of Oral Biomedicine Ministry of Education School & Hospital of Stomatology Wuhan University Wuhan China; ^2^ Department of Oral & Maxillofacial Head Neck Oncology School & Hospital of Stomatology Wuhan University Wuhan China; ^3^ Personalized Drug Therapy Key Laboratory of Sichuan Province Department of Pharmacy School of Medicine Sichuan Provincial People's Hospital University of Electronic Science and Technology of China Chengdu China; ^4^ Sichuan Provincial Key Laboratory for Human Disease Gene Study and Department of Laboratory Medicine Sichuan Provincial People's Hospital University of Electronic Science and Technology of China Chengdu China

**Keywords:** cancer hallmarks, cancer treatment, STAT3 pathway inhibitors, STAT3

## Abstract

Signal transducer and activator of transcription 3 (STAT3), a member of the STAT family, discovered in the cytoplasm of almost all types of mammalian cells, plays a significant role in biological functions. The duration of STAT3 activation in normal tissues is a transient event and is strictly regulated. However, in cancer tissues, STAT3 is activated in an aberrant manner and is induced by certain cytokines. The continuous activation of STAT3 regulates the expression of downstream proteins associated with the formation, progression, and metastasis of cancers. Thus, elucidating the mechanisms of STAT3 regulation and designing inhibitors targeting the STAT3 pathway are considered promising strategies for cancer treatment. This review aims to introduce the history, research advances, and prospects concerning the STAT3 pathway in cancer. We review the mechanisms of STAT3 pathway regulation and the consequent cancer hallmarks associated with tumor biology that are induced by the STAT3 pathway. Moreover, we summarize the emerging development of inhibitors that target the STAT3 pathway and novel drug delivery systems for delivering these inhibitors. The barriers against targeting the STAT3 pathway, the focus of future research on promising targets in the STAT3 pathway, and our perspective on the overall utility of STAT3 pathway inhibitors in cancer treatment are also discussed.

## Graphical abstract

Since the discovery of the signal transducer and activator of transcription 3 (STAT3) in 1994, the STAT3 pathway has been proven to play a pivotal role in cancer initiation, progression, and metastasis. This review summarizes the mechanisms of the STAT3 pathway regulation and the relationship between the STAT3 pathway and cancer hallmarks. The emerging development of the STAT3 pathway inhibitors, novel drug delivery systems, and perspective on the overall utility of the STAT3 pathway are also introduced.

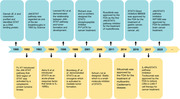

## 1 INTRODUCTION

The incidence and mortality rates of cancer are rapidly increasing worldwide. An estimated 19.3 million new cancer cases and 10.0 million cancer‐related deaths were recorded around the world in 2020. By 2040, the global cancer burden is expected to undergo a 47% rise from 2020.^1^ According to World Health Statistics 2021 posted by the World Health Organization, there were 1.9 million new cases diagnosed in 2021. Even in high‐income countries, cancer has been regarded as one of the leading causes of “premature death” defined as the death between the ages of 30 and 70.^2^ Currently, most patients with cancer are treated with surgery combined with radiotherapy, chemotherapy, and/or immunotherapy, depending on the types of cancer.^3^, ^4^, ^5^ Although these conventional therapies may achieve the significant tumor elimination effect initially, various barriers such as surgical trauma, high cost, drug resistance, and cytotoxicity in normal tissues are associated with the risk of treatment discontinuation and cancer recurrence and metastasis.^6^, ^7^, ^8^ Therefore, searching for new therapeutic targets and treatment methods are crucial for improving the survival rate and quality of life of patients with cancer.

Signal transducer and activator of transcription (STAT) proteins are latent cytoplasmic transcription factors comprising seven members, including STAT1, STAT2, STAT3, STAT4, STAT5a, STAT5b, and STAT6.^9^ Among them, STAT3 is involved in various basic cell functions including cell growth, survival, differentiation, regeneration, immune response, and cellular respiration. STAT3 can be strictly regulated by upstream signaling molecules, such as Janus kinase (JAK) and epidermal growth factor receptor (EGFR). It then localizes to the nucleus of cells and binds to target DNA to regulate the expression of downstream proteins.^10^, ^11^, ^12^, ^13^, ^14^ However, in addition to its functions in the normal cells, STAT3 activation in the tumor microenvironment (TME) is regarded as an oncogenic event. High phospho‐STAT3 expression is associated with poor prognosis in patients with various types of cancers such as non‐small cell lung cancer, gastric cancer, and colorectal cancer.^15^, ^16^, ^17^ Constitutive activation of STAT3 plays an important role in tumor formation, development, metastasis, and recurrence. These are strongly associated with cancer hallmarks and lead to poor patient outcomes. Therefore, the STAT3 pathway is a promising target for cancer therapy.^18^, ^19^, ^20^, ^21^, ^22^

In this review, we summarize the latest advances in STAT3 pathway in cancer. The mechanisms of STAT3 pathway regulation and the cancer hallmarks induced by the STAT3 pathway are also reviewed. We introduce the STAT3 pathway inhibitors and the novel drug delivery systems in preclinical studies and clinical trials for cancer treatment. Furthermore, the difficulties and prospects of targeting the STAT3 pathway in cancer are also discussed.

## 2 HISTORY OF STAT3 PATHWAY INVESTIGATION

**FIGURE 1:**
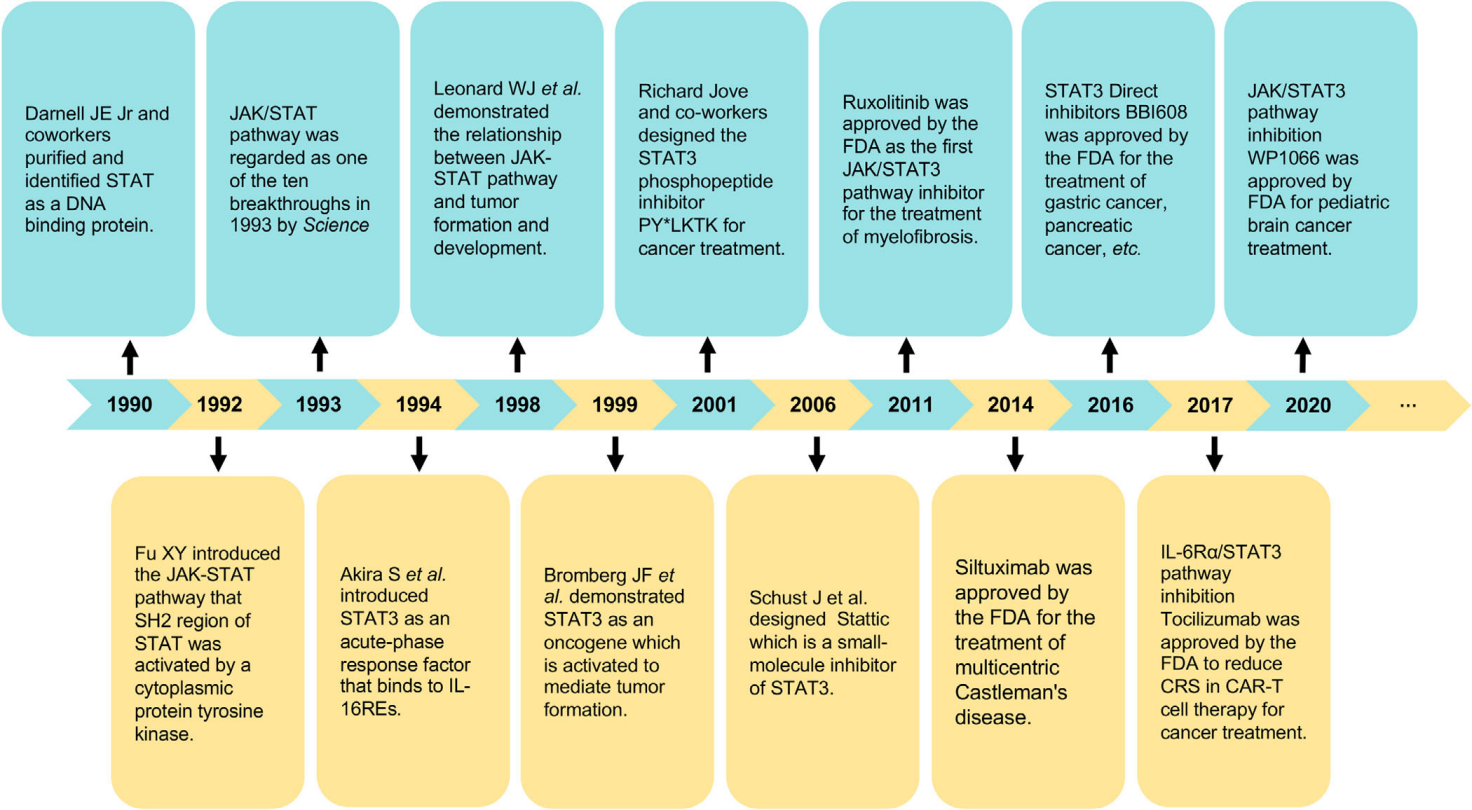
Brief history of signal transducer and activator of transcription 3 (STAT3) studies

**FIGURE 2:**
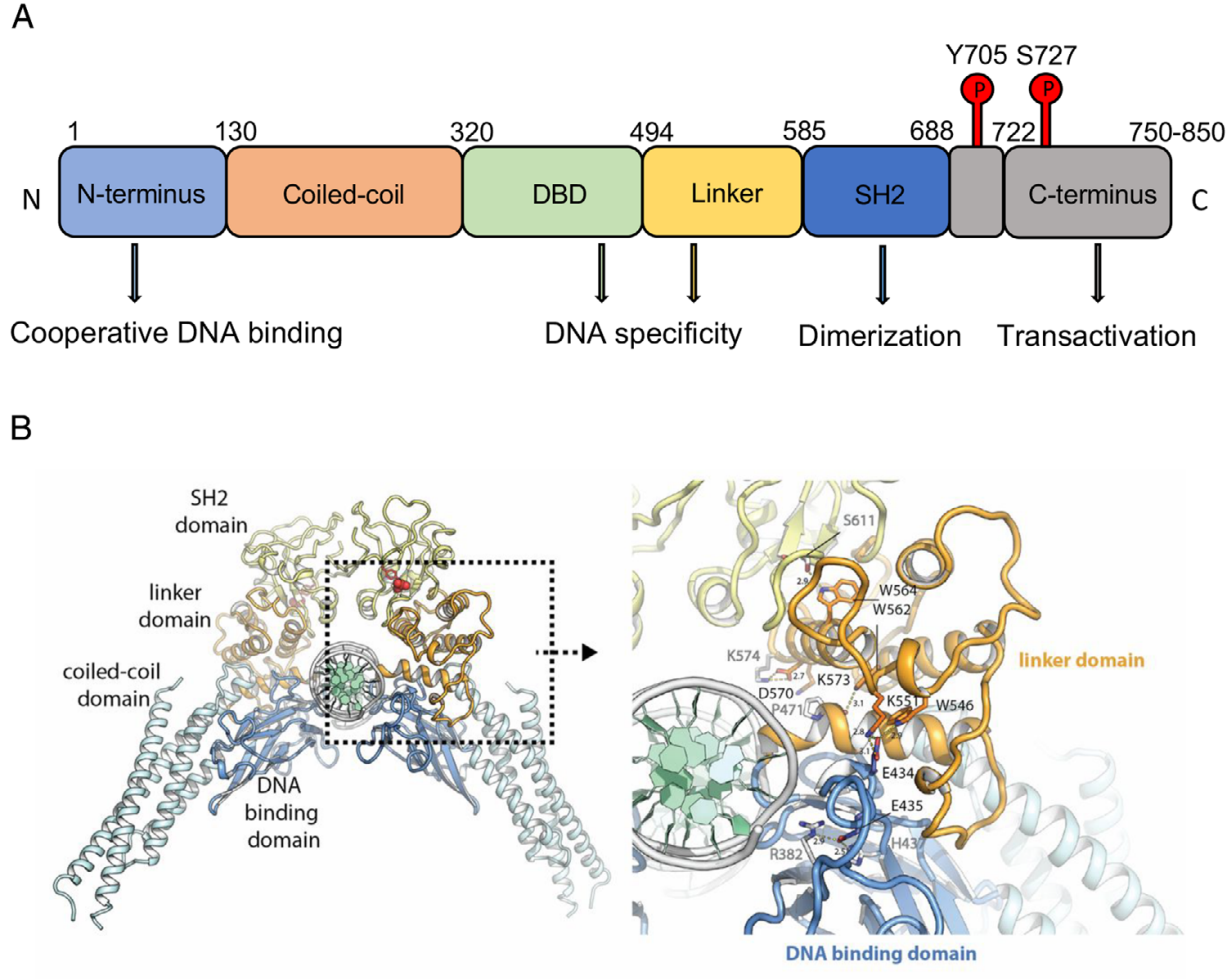
(A, B) Schematic and functional architecture of STAT3 protein. STAT3 has the construction, including N‐terminus domain, coiled‐coil domain, DNA‐binding domain, linker, SRC homology 2 and C‐terminus domain. Each of them plays a role such as DNA binding, dimerization, and transactivation. Reprinted with permission from Mertens et al.^28^ Copyright 2015 by National Academy of Sciences

In 1990, interferon‐stimulated gene factor 3 (ISGF3), the STAT protein complex activated by interferon α (IFNα), was purified and identified as a DNA‐binding protein by Darnell et al. (Figure 1).^23^ In 1992, Fu reported that the 113, 91, and 84 kDa (ISGF3α) proteins of the IFNα‐induced primary transcription factor ISGF3 had conserved Src homology domains (SH2 and SH3). IFNα treatment stimulates the activating phosphorylation of ISGF3α by protein tyrosine kinase (PTK), which facilitates direct signal transduction from the cell membrane to the nucleus.^24^ The discovery of JAK/STAT pathway was considered as one of the top 10 breakthroughs in 1993 by *Science*. Subsequently, an acute‐phase response factor (APRF) was observed to bind to interleukin‐6 (IL‐6) responsive elements and was activated in the cytoplasm to achieve the IL‐6 signal transduction.^25^ After the purification and cloning of APRF, Akira et al. demonstrated that APRF is highly homologous to the ISGF3 family and is involved in the glycoprotein 130 (gp130)‐mediated signaling pathway. And this APRF was regarded as the novel transcription factor named STAT3.^26^ The structure of STAT3 contains several conserved domains, including an N‐terminal domain, coiled‐coil domain, DNA‐binding domain (DBD), linker domain, SH2 domain, and C‐terminal domain, which are critical for its functions (Figure 2A,B).^27^, ^28^ The N‐terminal domain mediates tetramerization of two phosphorylated STAT3 dimers, dimerization of unphosphorylated STAT3, and binds other proteins to form functional complexes. These three main functions are associated with DNA binding, nuclear accumulation, and gene regulation of STAT3.^29^ The coiled‐coil domain is essential for early events in STAT3 signal transduction, including STAT3 recruitment for the receptor binding, tyrosine phosphorylation, dimerization, and DNA binding.^30^ The C‐terminal domain, also known as the trans‐activation domain (TDA), contains a specific tyrosine residue (Tyr705) that can be phosphorylated through the interaction of ligands with their receptors. STAT3 has an alpha‐helical linker domain spanning amino acid residues 500 to 575 that is followed by a classical SH2 domain. The SH2 domain is the highly conserved region that interacts with specific phosphotyrosine motifs of cytoplasmic signaling molecules and plays an important role in the dimerization of two STAT3.^31^

In 1999, Darnell et al. identified *STAT3* as an oncogene that could be constitutively activated and cause growth dysregulation in human tumor samples.^32^ Thus, STAT3 inhibitors have been developed for a long time. In 2001, Turkson et al. designed a STAT3 inhibitor, the SH2 domain‐binding phosphopeptide PY*LKTK, based on the key structural features of the STAT3 protein. PY*LKTK can bind to STAT3 in a manner similar to the Tyr(P)‐SH2 interaction, block STAT3 dimerization, and selectively inhibits STAT3 activation.^33^ Subsequently, the first non‐peptidic small‐molecule STAT3 inhibitor, Stattic, was developed. Stattic was reported to inhibit both the activation and dimerization of STAT3 by selectively inhibiting the binding of tyrosine‐phosphorylated peptides to the STAT3 SH2 domain.^34^ Moreover, the orally available direct STAT3 inhibitor napabucasin (BBI608) was approved by the United States Food and Drug Administration (FDA) for the treatment of two cancers, including gastric/gastroesophageal junction (GEJ) cancer and pancreatic cancer in 2016. In 2017 and 2020, the FDA approved tocilizumab and WP1066, respectively, to reduce cytokine release syndrome (CRS) induced by chimeric antigen receptor T (CAR‐T) cell therapy and to treat pediatric brain cancer. These results indicate that the STAT3 pathway is indispensable for treatment strategies against cancer in the past, present, and future.

## 3 *STAT3* AS AN ONCOGENE

Oncogenes are regarded as the viral or mutated cellular genes that play a decisive role in tumor formation. Activated oncogenes, such as K‐*ras* and *src*, inducing malignant transformation in tumor cells were found.^35^, ^36^ Generally, *STAT3* is considered an oncogene because of the following reasons.

Aberrant phosphorylation of STAT3 accumulates in nearly 70% of cancers, such as non‐small cell lung cancer and breast tumor. In human breast tissues, the level of STAT3‐binding activity was significantly higher in carcinomas than in normal and benign lesions.^37^ And STAT3 expression is 10.6‐fold higher in head and neck squamous cell carcinoma (HNSCC) tissues, compared to normal mucosa tissues derived from non‐HNSCC patients, leading to that activated *STAT3* is regarded as an oncogene.^38^, ^39^, ^40^, ^41^, ^42^

In addition to STAT3 overexpression in tumor tissues, cells bearing the activated STAT3 were transformed to form tumors in nude mice. Bromberg et al. replaced the C‐terminal loop with two cysteine residues in the SH2 domain of STAT3 to develop a STAT3 molecule (STAT3‐C) that can dimerize spontaneously without tyrosine phosphorylation. After transfection of immortalized fibroblasts with STAT3‐C, colonies were observed in soft agar, and tumors were present in nude mice 2–4 weeks after the injection of STAT3‐C clones.^32^

Aberrantly, phosphorylated STAT3 is also involved in tumor formation, development, and metastasis, which would influence the clinical outcome of patients. Lin et al. obtained tumor samples from 90 patients with glioblastoma (GBM) to examine the association between p‐STAT3 expression levels and patient outcomes. Patients with a large percentage of p‐STAT3 positive tumor cells had shorter progression‐free survival years and overall survival years. In this study, p‐STAT3 expression was considered an independent prognostic indicator in the outcome of patients with GBM.^43^ Fei et al. reported that high plasma STAT3 levels with the high programmed death ligand 1 (PD‐L1) expression induced by STAT3 led to the worst overall survival in patients with diffuse large B‐cell lymphoma.^44^

Moreover, STAT3 pathway inhibitors are reported to be effective in cancer treatment. In 2000, Song et al. proposed that targeting STAT3 can inhibit tumor growth through downregulating Bcl‐xL expression to increase apoptosis of HNSCC cancer cells, which supports the view of *STAT3* as an oncogene.^45^ Moreover, there is a significant correlation between downstream proteins regulated by STAT3 and lymph node metastasis, cancer stages, recurrence, and death in patients with lingual squamous cell carcinoma. Knocking down the upstream molecule of STAT3 or inhibiting STAT3 expression can decrease the growth and metastasis of HNSCC.^18^, ^46^, ^47^ The enhancer of zeste homolog 2/STAT3 signaling is demonstrated to play a promoting role in chemoresistance and chemo‐related adverse events in prostate cancer treatment. Targeting this signaling can significantly block the neuroendocrine differentiation in prostate cancer and inhibit the growth of chemoresistant cancer in vivo.^48^, ^49^ Collectively, the above studies provide evidence that the high expression level and constitutive activation of STAT3 in the TME are strongly associated with cancer formation and poor prognosis of patients.

However, some researchers hold the converse view that *STAT3* is an oncosuppressor. Kenichi Shinagawa et al. showed that the clinicopathological factors are significantly associated with IL‐6 expression rather than STAT3 expression levels. The expression of IL‐6 and STAT3 in 116 patients with oral squamous cell carcinoma (OSCC) were independent, and only high IL‐6 expression significantly promoted vascular invasion and decreased the 5‐year disease‐free survival rate.^50^ Moreover, STAT3‐positive cells are associated with favorable outcomes in some cancers. High nuclear STAT3 levels were associated with a 72.4% overall survival rate in patients with HNSCC at 5 years, compared to 38.3% in the low nuclear STAT3‐level group. High STAT3 expression may contribute to the early stages of tumor formation and development by inhibiting apoptosis of tumor cells.^51^ Tissue microarray‐based study of both node‐negative and node‐positive breast tumors showed that overexpression of phospho‐STAT3 (Tyr705) improved the short‐term survival and long‐term survival of patients with breast tumor, which indicated a better prognosis of patients with STAT3 positive breast cancer.^52^, ^53^ Pencik et al. reported that the ARF–Mdm2–p53 tumor suppressor axis is regulated by STAT3, and loss of STAT3 signal transduction increases the risk of prostate cancer metastasis and recurrence in mouse models, indicating a poor outcome after treatment with IL‐6/STAT3 inhibitors.^48^, ^54^

## 4 REGULATORS OF THE STAT3 PATHWAY

### 4.1 Activation of STAT3

STAT family proteins were first reported in the last three decades as the transcription factors that are activated in a ligand‐dependent manner and promote the rapid induction of gene expression.^55^, ^56^, ^57^ It is reported that more than 40 different polypeptide ligands, including cytokines, JAK kinases, and growth factors, are associated with STAT phosphorylation. As one of the most important members of the STAT family, STAT3 is activated through tyrosine phosphorylation in response to epidermal growth factor (EGF) and IL‐6 and others to play an important role in regulating cell proliferation, differentiation, and apoptosis after DNA binding.^57^, ^58^ Once activated by JAK, STAT3 molecules start dimerizing mainly via the SH2 domain with the help of TDAs to form the reciprocal pY‐SH2 interactions. Then, phosphorylated STAT3 can translocate into the nucleus and bind to specific DNA to activate the downstream proteins gene expression.^56^, ^59^, ^60^

### 4.2 Positive regulators

**FIGURE 3:**
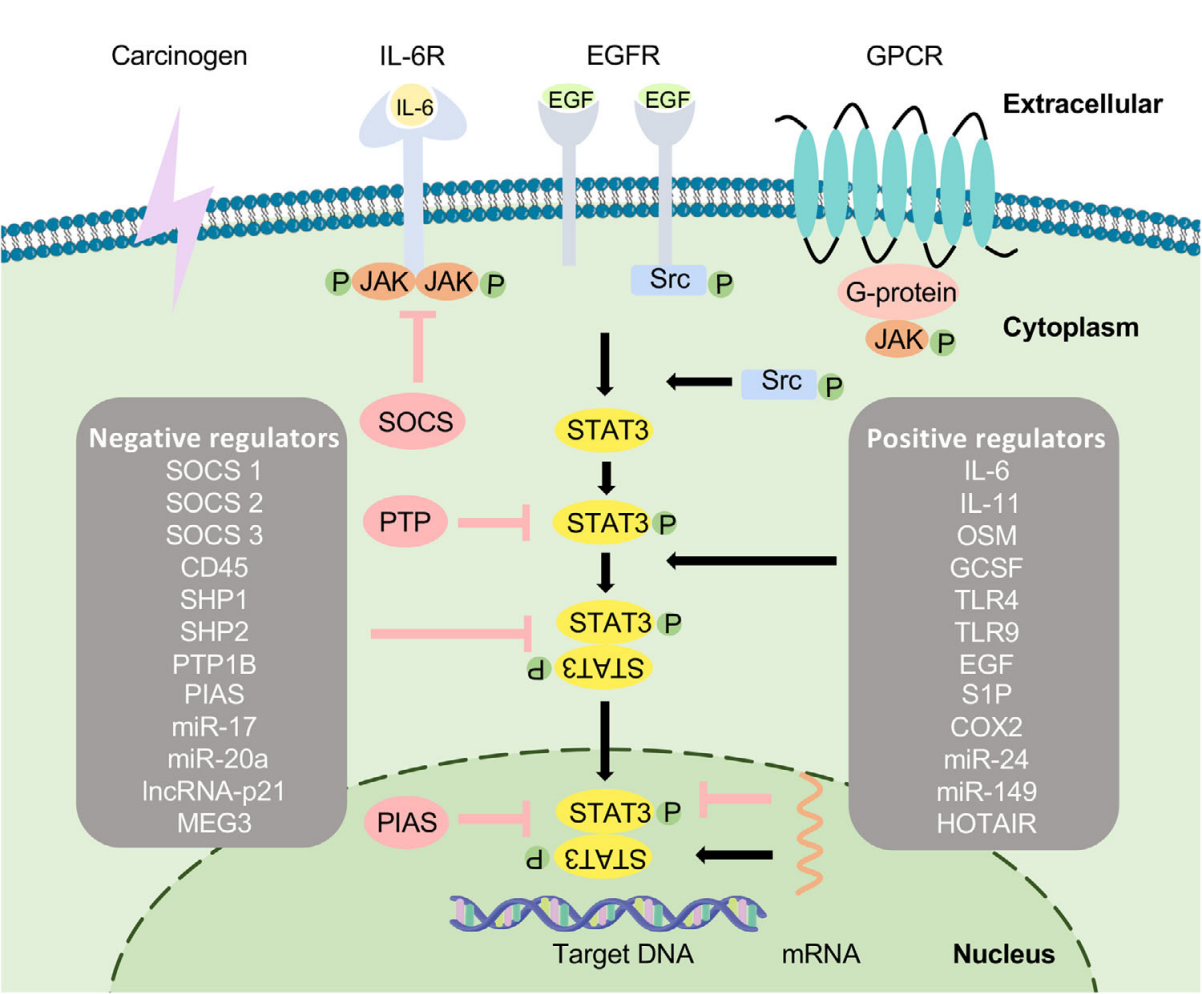
Schematic of pathways activating STAT3 signaling. Once cytokines and growth factors such as interleukin‐6 (IL‐6), epidermal growth factor (EGF) and G‐proteins as positive regulators bind to their receptors, ligand‐bound receptors undergo conformational changes and activate Janus kinase (JAK) family. STAT3 molecules are phosphorylated on Y705 by intracellular non‐receptor tyrosine kinases (RTKs) such as JAK and RTKs such as EGF receptor, or STAT3 can also be activated directly by Src and Abl. While PTP, suppressor of cytokine signaling and protein inhibitor of activated STAT as the negative regulators can inhibit the activity of STAT3. The dimerization of two activated STAT3 molecules binding via SH2 domain enters the nucleus and then binds to target gene

As a transcription factor, STAT3 is expressed in almost all types of cells and is tightly activated by upstream regulatory molecules under normal conditions. However, the continuous release of upstream molecules causes the constitutive activation of STAT3 in the TME (Figure 3). JAK/STAT pathway is an important oncogenic signaling cascade activated by multiple adaptor proteins such as IL‐6, EGF, and IFN‐γ.^61^ Persistent activation of STAT3 induced by cytokines and growth factors is associated with cell proliferation, differentiation, and apoptosis in cancer.^62^, ^63^ IL‐6 is expressed at the high levels in the TME by immune cells, tumor cells, and stromal cells and acts as both pro‐ and anti‐inflammatory cytokine. IL‐6 binds to IL‐6R on the cell membrane and subsequently forms a protein complex with IL‐6Rβ (gp130 receptor), mediates the activation of STAT3, and promotes tumorigenesis.^64^, ^65^, ^66^ IL‐6 signaling can also be mediated by a trans‐signaling pathway, wherein the interaction of IL‐6 with the secreted from IL‐6R (sIL‐6R) can form the IL‐6–sIL‐6R complex and subsequently bind to gp130.^67^ Moreover, activated STAT3 can induce IL‐6 expression to generate a positive‐feedback loop for STAT3 overexpression. The JAK family contains four non‐receptor tyrosine kinases (RTKs) including JAK1, JAK2, JAK3, and TYK2. Except JAK3 expressed in hematopoietic cells, others are widely expressed in various cells. With the engagement of gp130, JAK is activated to phosphorylate STAT3 subsequently.^68^

In addition to the IL‐6/JAK/STAT3 pathway, the activation of EGFR, fibroblast growth factor receptor, C‐X‐C chemokine receptor (CXCR), G‐protein coupled receptor, and B7‐H3, as well as the stimulation with carcinogenic (areca nut extract) and others, can also cause STAT3 phosphorylation via the JAK/STAT3 pathway.^69^, ^70^, ^71^, ^72^, ^73^ It was reported that other classes of non‐receptor PTKs could stimulate STAT3 activation. The Src family of kinases, including Src, Lck, Hck, Lyn, Fyn, and Fgr can mediate STAT3 activation. Among them, viral Src was reported to induce constitutive STAT3 activation in a JAK‐independent manner. Cellular Src tyrosine kinase was later demonstrated to be a positive regulator of STAT3 activation via stimulation with platelet‐derived growth factor and ligand of the human EGFR family.^74^, ^75^, ^76^ EGFR, a receptor PTK, is upregulated in most epithelial cancers, and EGFR signaling contributes to cancer cell proliferation and survival. Activated EGFR can directly interact with SH2 domains to phosphorylate STAT3. Moreover, treatment by targeting both EGFR and STAT3 was demonstrated to play an important role in EGFR–STAT3 feedback loop blockade and restriction of pancreatic cancer volume.^77^

Long non‐coding RNAs (lncRNAs) with a length of more than 200 nucleotides can induce or reduce STAT3 expression.^78^ MicroRNA (miRNA), as one of the non‐coding endogenous RNA, can also induce or reduce STAT3 expression by regulating mRNA degradation or translational inhibition via binding to targeting mRNA molecules.^79^, ^80^ Plenty of studies have found that lncRNAs such as HOX Transcript Antisense RNA (HOTAIR), Gastric Cancer Associated Transcript 3 (GACAT3), Nuclear Paraspeckle Assembly Transcript 1(NEAT1), Forkhead Box D2 Adjacent Opposite Strand RNA 1 (FOXD2‐AS1), and Inter‐Alpha‐Trypsin Inhibitor Heavy Chain 4 Antisense RNA 1 (ITIH4‐AS1), as well as miRNAs including miR‐24, miR‐629, miR‐149, miR‐495‐3p, and miR‐34a can act as the positive regulators of the STAT3 pathway.^81^, ^82^, ^83^, ^84^, ^85^, ^86^

### 4.3 Negative regulators

STAT3 is temporarily activated and tightly regulated by negative regulators that can silence the STAT3 pathway in normal tissues. However, these negative regulators are inhibited in tumor cells. These negative regulators either block the STAT3 signaling pathway or directly act on the STAT3 protein and, thus, may inspire new ideas for cancer treatment (Figure 3).

Due to the significance of tyrosine phosphorylation in STAT3 activation, tyrosine phosphatases can negatively regulate STAT3.^87^ The protein tyrosine phosphatase (PTP) family, which includes PTP receptor‐type D (PTPRD), PTP receptor‐type T, PTP receptor‐type K, SH2‐domain‐containing PTP1 (SHP1), SHP2, PTP‐non‐receptor type 9, and T‐cell PTP, is essential for the negative regulation of JAK‐STAT3 signaling through STAT3 dephosphorylation.^88^, ^89^, ^90^, ^91^, ^92^, ^93^ Pertinently, PTPRD is downregulated in nasopharyngeal carcinoma (NPC), and PTPRD overexpression can promote the sensitivity of NPC cells to radiotherapy owing to STAT3 dephosphorylation.^94^ Gupta et al. reported that morin‐induced SHP1 expression abrogated the effect of STAT3 and promoted chemo‐sensitization of HNSCC cells.^88^

The suppressor of cytokine signaling (SOCS) proteins family has eight members including SOCS 1–7 and cytokine‐inducible SH2‐containing protein. Each of them inhibits STAT3 phosphorylation via different mechanisms: directly binding its kinase inhibitory region and SH2 domains to JAK kinase domain to block STAT3 phosphorylation; binding its SH2 domain to JAK‐phosphorylated cytokine receptors to prevent STAT3 recruitment.^95^ Considering SOCS1, Yoshikawa et al. showed that silencing of SOCS1 induced the constitutive activation of the JAK2/STAT3 pathway in hepatocellular carcinoma (HCC). The growth of SOCS1 restoration cancer was suppressed with the same efficacy as treatment with the JAK2 inhibitor AG490, which indicated the negative regulation of SOCS in the JAK/STAT3 pathway.^96^ Moreover, SOCS1 expression inhibits the growth and metastasis of prostate cancer by decreasing levels of cyclins D1 and E and cyclin‐dependent kinases (CDK) 2 and 4.^97^

Activated STAT3 is also regulated in the nucleus to regulate gene expression. It was reported that the protein inhibitor of activated STAT (PIAS) can bind to activated STAT dimers and prevent them from binding to DNA.^98^ The most important negative regulator in the PIAS family is PIAS3, and many studies demonstrated that upregulation of PIAS3 expression can inhibit cell proliferation and increase drug chemosensitivity in various tumors.^99^, ^100^, ^101^ Jiang et al. identified that JAK/STAT hyperactivation induced by SOCS3 and PIAS3 deficiency is associated with the development of early‐stage myeloid‐derived suppressor cells (eMDSCs) and immunosuppression in breast cancer.^102^

Moreover, various lncRNAs were demonstrated to induce the STAT3 gene expression and were downregulated in cancer tissues, which were associated with the tumor progression and poor prognosis. This negative correlation between STAT3 and specific lncRNAs indicated that lncRNAs could work as the negative regulators of STAT3 pathway.^103^, ^104^, ^105^ It was reported that lncRNAs such as lncRNA‐p21, MEG3, and PTCSC3, as well as miRNAs such as miR‐548d‐3p and miR‐17 cluster family members, can directly target STAT3 and thereby regulate E‐cadherin expression.^103^, ^106^, ^107^, ^108^, ^109^, ^110^

## 5 REGULATION OF CANCER HALLMARKS BY THE STAT3 PATHWAY

**FIGURE 4:**
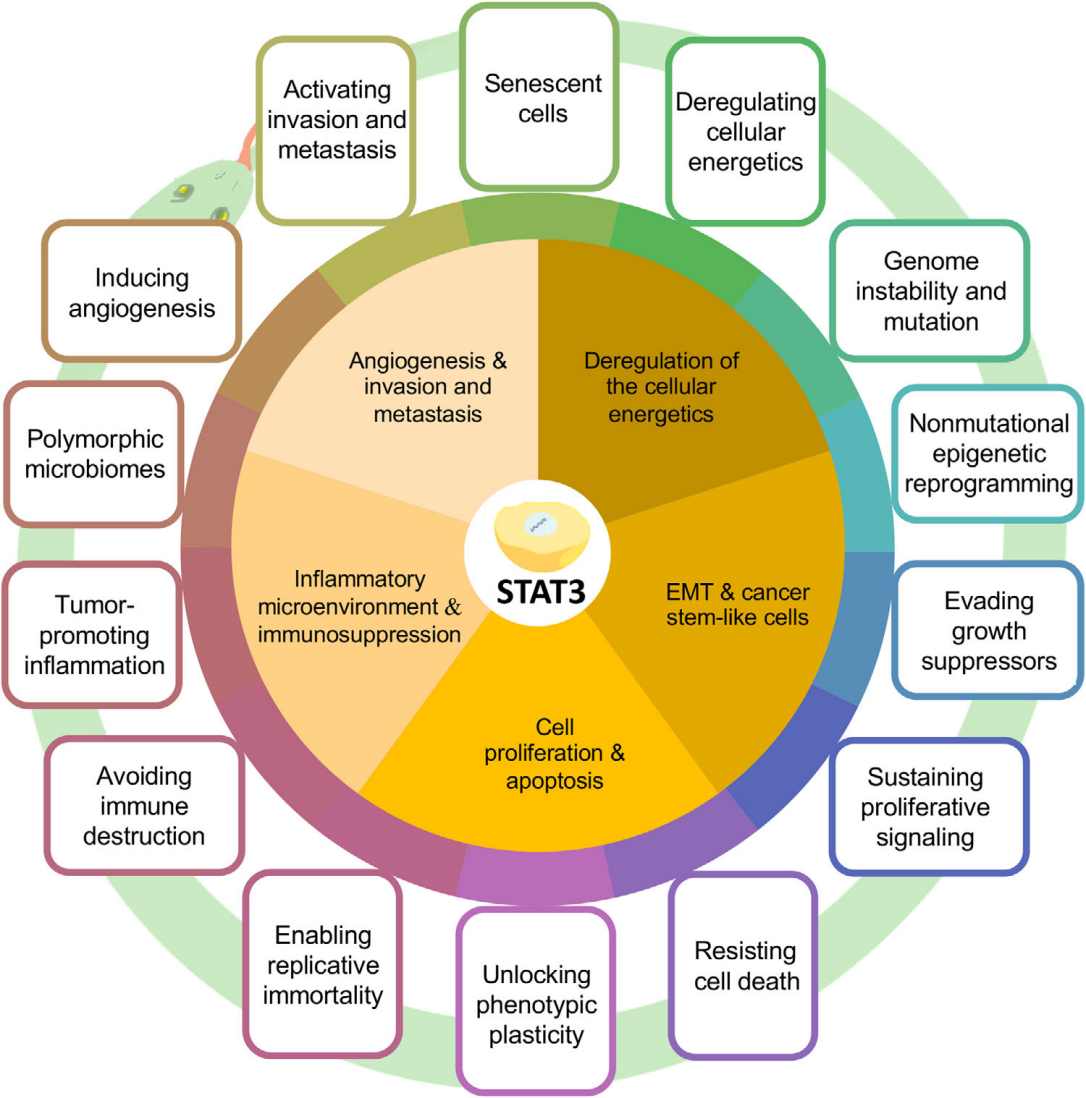
Schematic of the relationship between STAT3 and cancer hallmarks. STAT3 improve the representation of tumor hallmarks through the following five aspects: inflammatory microenvironment and immunosuppression; cell proliferation and apoptosis; epithelial‐mesenchymal transition and cancer stem‐like cells; deregulation of the cellular energetics; angiogenesis and invasion and metastasis

In 2000, Hanahan et al. proposed hallmarks of cancer, which contained six essential alterations inducing malignant cell growth.^111^ Later in 2011, because of the advancement in cancer research, “energy metabolism” and “immune suppression” were also considered cancer hallmarks. A new generation of cancer hallmarks has been enumerated and includes “sustaining proliferative signaling,” “evading growth suppressors,” “avoiding immune destruction,” “enabling replicative immortality,” “tumor‐promoting inflammation,” “activating invasion and metastasis,” “inducing angiogenesis,” “genome instability and mutation,” “resisting cell death,” and “deregulating cellular energetics.”^112^ Recently, the number of cancer hallmarks increased from 10 to 14. “Unlocking phenotypic plasticity,” “senescent cells,” “non‐mutational epigenetic reprogramming,” and “polymorphic microbiomes” were considered as the emerging cancer hallmarks.^113^ STAT3, as a multifunctional regulator in cancer formation, development, and metastasis, can influence all cancer hallmarks through the following five ways (Figure 4).

### 5.1 Cell proliferation and apoptosis

Cancer is considered a cell cycle disease, and many studies suggest that connections between oncogenes and an abnormal cell cycle are caused by mutations or abnormalities in the expression of cyclins and cyclin‐CDKs in a variety of cancers.^114^, ^115^, ^116^ The activated cyclin‐CDK complex formed by the binding of the cyclin box from cyclins with a well‐conserved family of protein kinases regulates the progression of cells through the division cycle.^114^ Cyclin D1 (CCND1) protein, which is one of the major isoforms of D‐type cyclins, can interact with CDK4/6 and accelerate the progression of the cell cycle through the G1 phase. CCND1 was reported to be overexpressed and accumulated in association with p‐STAT3 in gastric and oral cancer cells. Moreover, after treatment with STAT3 pathway inhibitors, cyclin expression was significantly reduced.^117^, ^118^ Luo et al. showed that upregulation of CCND1 by STAT3 enhanced the proliferation of gastric cancer cells. JAK/STAT3 blockade by AG490 could significantly decrease the CCND1 protein levels to inhibit cancer cells proliferation.^118^ Yang et al. reported that icaritin can be used to inhibit OSCC cell proliferation via the regulation of the the STAT3 pathway.^119^

Additionally, the STAT3 pathway is reported to promote cell survival and inhibit apoptosis by modulating the apoptotic regulatory proteins including Bcl‐2 family.^45^, ^120^ As regulator of programmed cell death, Bcl‐2 family members are categorized into three subfamilies including prosurvival proteins, BH3‐only proapoptotic proteins and multidomain proapoptotic proteins. Cell apoptosis can be evaluated through the expression ratio of proapoptotic to prosurvival factors.^121^ Pro‐survival proteins such as Bcl‐X_L_, Bcl‐2, and MCL‐1 are overexpressed in many cancers and contribute to tumor initiation, progression, and therapeutic resistance.^122^, ^123^, ^124^ Catlett‐Falcone et al. demonstrated that the growth and survival of multiple myeloma depended on IL‐6 receptor signaling, which is associated with STAT3‐induced Bcl‐X_L_ expression. Consequently, blockade of gene regulation mediated by STAT3 could inhibit Bcl‐X_L_ expression and induce apoptosis in cancer cells.^125^ Oh et al. used licochalconce (LC) H for OSCC treatment, and their results showed that the JAK2/STAT3 pathway and STAT3 target genes such as Bcl‐2 were suppressed by LCH, which caused the cell apoptosis and inhibited cell proliferation and colony formation in OSCC cells.^126^

In addition to cancer cells, STAT3 expression in senescent cancer‐associated fibroblasts (CAFs) can improve the viability of cancer cells, which is associated with the senescence‐associated secretory phenotype. Yasuda et al. demonstrated that the senescence of CAFs induced by pro‐inflammatory cytokines was blocked after the treatment with JAK/STAT3 pathway inhibitors. The JAK/STAT3 inhibitor effectively blocks the peritoneal tumor formation in a mouse model.^127^ Thus, constitutive activation of STAT3 can upregulate the expression of anti‐apoptotic proteins and cyclins to regulate cell cycle, promote cell proliferation, and inhibit cell apoptosis.

### 5.2 Chronic inflammatory microenvironment and immunosuppression

Tumors are also considered “wounds that do not heal,” and many similarities can be found between the microenvironment of chronic inflammatory conditions and tumors. Chemokines and cytokines in TME interact with cancer cells.^20^, ^128^ Dynamic participants in the TME, including tumor‐associated macrophages (TAMs), neutrophils, dendritic cells, and regulatory T‐cells (Tregs) induce an immunosuppressive TME and enhance the evasion of tumor from immune surveillance, which are associated with cancer progression.^129^, ^130^, ^131^ PD‐L1, an immune checkpoint, plays an important role in delivering pro‐survival signals to cancer cells to protect them from tumor‐specific immunity.^132^ The over‐expressions of PD‐L1 in cancer cells and PD‐1 in stromal cells were reported to be associated with the phosphorylation of STAT3, which demonstrated the immunosuppressive roles of the STAT3 pathway.^133^ Xu et al. observed an effective cytotoxicity of natural killer (NK) cells after blockade of the IL‐6/JAK/STAT3 pathway and PD‐L1, which indicated that NK cell‐mediated recognition and cytotoxicity against tumor cells were inhibited via STAT3 pathway‐induced PD‐L1 expression in tumor cells.^134^ In addition to tumor cells, PD‐L1 is also overexpressed via the STAT3 pathway on the surface of neutrophils with the engagement of PD‐1 to block the activation of T‐cells and NK cells. CAFs derived from HCC are reported to protect neutrophils from apoptosis and promote neutrophils activation via the IL‐6/JAK/STAT3 pathway. Once activated, neutrophils inhibit T‐cell immunity through the STAT3/PD‐L1 axis.^135^ Tumor‐derived granulocyte colony stimulating factor (G‐CSF) can also activate the STAT3 pathway in neutrophils and show pro‐tumor effects involving dysfunction of NK cells via PD‐L1/PD‐1 interactions.^136^

Macrophages, which are regarded as the tissue sentinels, can eliminate or repair damaged cells and matrices to maintain tissue integrity. After the stimulation with different activation programs, macrophages are polarized into two different modes with their own metabolic functions, including M1‐like macrophages that produce pro‐inflammatory cytokines and M2‐like macrophages that have anti‐inflammatory and wound healing characteristics.^137^ IL‐6‐dependent STAT3 activation induces M2 polarization while inhibiting M1 activation, and this promotes cancer progression and reduces patient survival.^138^, ^139^, ^140^ Besides inducing M2 polarization, the STAT3 pathway also upregulates PD‐L1 expression in cancer cells induced by TAMs. Zhang et al. conducted a study to demonstrate how TAMs suppress immune activation and promote the invasive capacity of cancer cells via the STAT3 pathway. The results showed that IFN‐γ produced by TAMs enhances the constitutive activation of STAT3 in cancer cells, which is associated with PD‐L1 expression.^141^ Simultaneously, MDSCs play an immunosuppressive role in many cancers, and IL‐10/STAT3 signaling in MDSCs suppresses CD8^+^ T‐cell proliferation and promotes the development of Tregs in tumors.^142^, ^143^, ^144^

**FIGURE 5:**
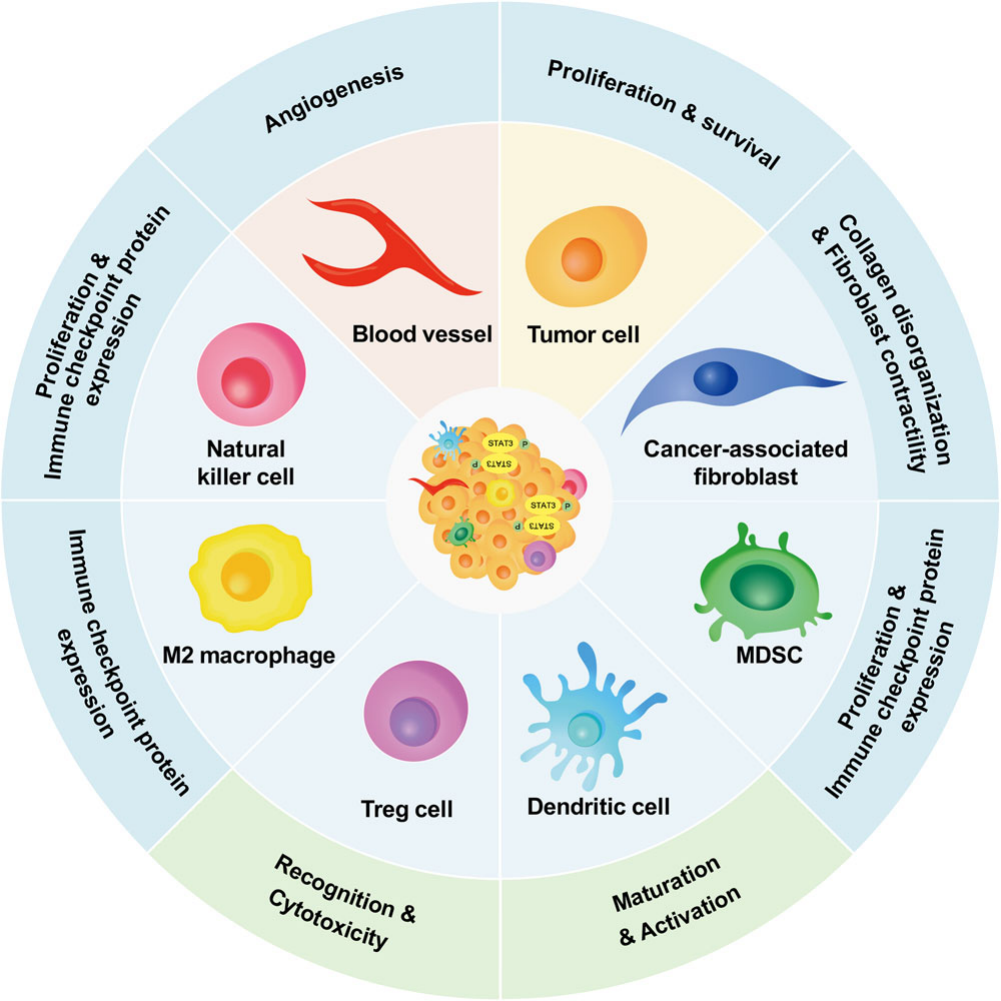
Schematic of STAT3 signaling in tumor microenvironment (TME). STAT3 activation has the ability in affecting TME via up‐ or downregulating downstream molecules and promoting tumor cell proliferation and survival, angiogenesis, immune evasion as the result. The functions of natural killer cell and dendritic cell in antigen presentation and target cell recognition are inhibited. While macrophage polarization toward M2‐like endotype and the immune checkpoint expression and proliferation of myeloid‐derived suppressor cell, cancer‐associated fibroblasts, and regulatory T‐cell are promoted by phosphorylated STAT3

Additionally, p‐STAT3 in the TME can induce the production of IL‐6, IL‐10, and vascular endothelial growth factor (VEGF) and transform dendritic cells (DCs) into regulatory DCs that contribute to immune tolerance.^20^, ^130^, ^145^ Kortylewski et al. ablated *STAT3* in hematopoietic cells of adult mice with the *Mx1‐Cre‐lox*P system via injection of poly(I:C), and the anti‐tumor immunity was evaluated. The results showed that *STAT3* deletion in hematopoietic cells enhanced the ability of DCs to present antigens and activate T‐cells, improved the ability of granulocytes and NKs to eliminate targeting tumor cells, increased T‐cell responses to tumor antigens, and inhibited tumor growth in tumor‐bearing mice.^146^ Constitutive STAT3 activation decreases the expression of T‐cell chemotactic factors (such as RANTES and IP‐10) to inhibit T‐cell chemotaxis. Further, S1PR1 promotes STAT3 activation in CD4^+^ T‐cell associated with Tregs accumulation at tumor sites.^147^, ^148^ However, some studies reported contradictory results. Hanna et al. showed that IL‐10R/STAT3 pathway correlated with the accumulation of a PD‐1^int^ CD8^+^ T‐cell subset, which plays an important role in tumor elimination, and the loss of IL‐10R/STAT3 signaling enhanced the accumulation of functionally impaired PD‐1^hi^ CD8^+^ T‐cells associated with the progression of tumors in a chronic lymphocytic leukemia model.^149^ Taken together, constitutive activation of STAT3 regulates the adaptive immune response in the chronic inflammatory TME and mediates immunosuppression in cancers (Figure 5).

### 5.3 Angiogenesis, invasion, and metastasis

Angiogenesis, which refers to the formation of new blood vessels, is activated by hypoxia and nutrient deprivation to meet the metabolic demands, to remove the waste products from solid tumors, and is involved in initiation, progression, and metastasis of cancer.^150^, ^151^ Tumor angiogenesis is mediated by tumor‐secreted angiogenic cytokines, growth factors, and integrins expressed both on angiogenic endothelium and tumor cells. Integrins can integrate signals between the extracellular matrix (ECM) and cellular and chemokines, such as CCL4, to coordinate the migration of circulating endothelial progenitor cells to the hypoxic area.^152^ VEGF is associated with vascular development and is regarded as one of the most important factors for the induction of angiogenesis. VEGF can be activated by hypoxic TME and directly upregulated by constitutive activation of STAT3.^153^, ^154^ STAT3 directly regulates VEGF expression via its promoter, and mutations in the STAT3 binding site in the VEGF promoter abrogate this regulation.^153^ VEGF expression can also be indirectly induced by the hypoxic TME through a STAT3/hypoxia‐inducible factor 1 alpha (HIF‐1α)‐dependent pathway. Hypoxia‐induced pSTAT3 accelerates the accumulation of HIF‐1α protein and prolongs its half‐life in solid tumor cells, which subsequently enhances VEGF expression.^155^, ^156^ The study by Liu et al. employing sulforaphane to block angiogenesis in HCC demonstrated this indirect mechanism. After sulforaphane treatment, the STAT3/HIF‐1α/VEGF pathway was blocked and the angiogenesis and tumor growth were inhibited.^157^ High expression levels of STAT3 and VEGF are also associated with lymph node involvement in esophageal squamous cell cancer, indicating that STAT3/VEGF pathway promotes cancer cell lymphatic metastasis and is correlated with pTNM stage.^158^

Moreover, ECM remodeling induced by matrix metalloproteinases (MMPs), which can degrade ECM components is also involved in tumor angiogenesis.^159^ In normal tissues, the function of MMPs is counteracted by tissue inhibitors of metalloproteinases (TIMPs) to maintain the balance between angiogenic and anti‐angiogenic effects. However, the effect of MMPs is increased in tumor tissues, which break this balance and promote angiogenesis by remodeling the basement membrane and promoting pericyte recruitment.^152^, ^160^ Overexpression of MMPs is also reported to be induced by pSTAT3. Considering MMP‐9, Roy et al. identified the mechanisms by which A Disintegrin and Metalloproteinase domain‐containing protein 12 (ADAM12) induces angiogenesis in breast cancer. The results showed that ADAM12‐regulated angiogenesis was correlated with the upregulation of the proangiogenic factors including VEGF and MMP‐9 and the anti‐angiogenic factors including TIMP2. The silencing of ADAM12 inhibited STAT3 activation, which demonstrated that angiogenesis can be promoted by activating MMP‐9 in a STAT3‐dependent manner.^161^ The elevated gene transcription of MMP‐9 was demonstrated to be associated with activation of the STAT3 in tumor‐associated myeloid cells.^162^ Additionally, MMP overexpression contributes to cancer cell invasion and metastasis, and IL‐8/STAT3 signaling in HNSCC can also upregulate the expression of MMP‐2 and ‐9 and play a key role in cancer invasion and metastasis.^163^, ^164^ Further, the migration of endothelial cells (ECs) during angiogenesis can be facilitated with the help of MMP‐2/9 overexpression. In addition to important features of STAT3 pathway‐related angiogenesis including cell migration, invasion, and tube formation, constitutive activation of STAT3 in CAFs and tumor cells can regulate collagen fibrogenesis and collagen disorganization and fibroblast contractility resulting in increased cancer invasion and metastasis.^20^, ^165^, ^166^ Thus, constitutive activation of STAT3 promotes angiogenesis, invasion, and metastasis of cancer cells.

### 5.4 Epithelial‐mesenchymal transition (EMT) and cancer stem‐like cells (CSCs)

EMT is a general phenomenon observed in embryonic development, tissue remodeling, and wound repair. However, the loss of intracellular adhesion and the transition from an epithelial to the mesenchymal phenotype are correlated with tumor progression and can be observed in cancer lesion area by the loss of E‐cadherin and gain of vimentin, N‐cadherin, and fibronectin.^167^, ^168^ In cancer tissues, the unlocking of the phenotypic plasticity of cancer cells concurs with EMT, and the EMT‐related regulators including STAT3 are associated with cancer cell dedifferentiation.^169^ Studies have demonstrated that downstream of STAT3, estrogen‐regulated zinc transporter LIV‐1, miR‐21, IL‐6, HIF, and VEGFR2 can repress E‐cadherin and modulate EMT in many cancers.^170^, ^171^, ^172^, ^173^, ^174^ Dysregulation of the STAT3 pathway in CAFs in pancreatic ductal adenocarcinoma (PDAC) samples was reported to be associated with transforming growth factor beta 1 secretion and the significant single‐cell population shifts toward EMT, leading to the aggressive behavior of PDAC.^175^

CSCs are a heterogeneous population of cancer cells that play an important role in cell self‐renewal and differentiation. CSCs are associated with tumor regeneration and metastasis and are considered indicators for the prognosis of patients with cancer.^176^, ^177^, ^178^ Activation of the STAT3‐dependent pathways including the STAT3/FAK/ERK pathway, STAT3/Oct‐4 pathway in various cancer cells, and the IL‐8/STAT3 pathway and EGFR/STAT3/Sox‐2 pathways in TAMs are shown to contribute to the cancer stemness.^179^, ^180^, ^181^, ^182^ Moreover, a link between EMT and CSCs is demonstrated.^183^, ^184^ The CSCs are associated with EMT exhibit and maintain EMT after cancer treatment. EMT phenotypes promote the metastatic proliferation of CSCs among cancer cells. This is crucial for treatment resistance and occurrence, progression, and recurrence of various tumor types.^151^, ^185^ STAT3 pathway is reported to regulate the EMT and CSC in cancer development. Oncostatin M (OSM), a crucial inducer of JAK/STAT3 pathway activation, promotes EMT and generates cells with CSC properties in a STAT3‐dependent manner.^186^, ^187^ To further explore the mechanism by which the OSM/STAT3 pathway induces EMT and CSCs, Junk et al. infected epithelial/non‐CSCs with retroviruses encoding shSMAD2, SMAD3, SMAD4 to inhibit SMAD proteins. SMAD3 knockdown significantly reduced mesenchymal/CSC expansion, compared to other treatment groups, which implicated the OSM/JAK/STAT3/SMAD3 pathway in EMT induction and CSCs generation.^188^ Recent studies also demonstrated that STAT3 activation is involved in cisplatin chemotherapy resistance through the STAT3/Snail pathway, which is associated with the EMT‐like phenotype and stem‐like properties acquisition. Besides, resistance to 5‐fluorouracil, temozolomide, and sorafenib treatment is also related to the enrichment of CSCs caused by p‐STAT3 upregulation.^189^, ^190^, ^191^, ^192^, ^193^ Thus, activated STAT3 can promote EMT and maintain the stemness of cancer stem cells, thereby affecting the prognosis of patients with cancer.

### 5.5 Deregulation of the cellular energetics

Cancer cells convert pyruvate into lactate irrespective of the presence of oxygen via aerobic glycolysis, also known as the “Warburg effect,” and metabolism in cancer cells can accelerate adenosine triphosphate (ATP) synthesis and maintain rapid cell proliferation.^194^ An increased glycolytic rate and concomitant limitation of the tricarboxylic acid cycle (TCA cycle), even in the presence of completely functional mitochondria and sufficient oxygen, typify the Warburg effect.^195^, ^196^ The mechanism underlying the Warburg effect and its association with cancer cell proliferation is not fully understood, but the STAT3 pathway is demonstrated to contribute to Warburg's vicious circle. Studies show that STAT3 activation is associated with the M2 isozyme of pyruvate kinase (PKM2)/HIF‐1α positive feedback loop and promotes proliferation of many types of cancer cells, such as human HCC cells, breast cancer cells, and colorectal cancer cells. PKM2 is typically present in tumor cells and can be activated by HIF‐1α in response to oxygen deprivation, cytokines, and oncogenes in the TME. Activated PKM2 can promote HIF gene transcription and STAT3 activation, and pSTAT3 can induce the expression of HIF‐1α and MEK5 and, thereby, enhance the Warburg effect.^197^, ^198^, ^199^, ^200^, ^201^ The expression of hexokinase 2 (HK2), which is accelerated by the STAT3 pathway, is also involved in the Warburg effect. Pu et al. showed that circCUL3 was upregulated in gastric cancer tissues and activated HK2 expression by targeting the miR‐515‐5p/STAT3 pathway. The results demonstrated molecular interactions between activated STAT3 and HK2.^202^ Thus, STAT3 signaling promotes the Warburg effect in cancer tissues by upregulating downstream glycolytic proteins; it also promotes cancer cell proliferation related to glycolysis through mechanisms that are not completely understood.

## 6 STAT3 AS A THERAPEUTIC TARGET IN CANCER TREATMENT

**FIGURE 6:**
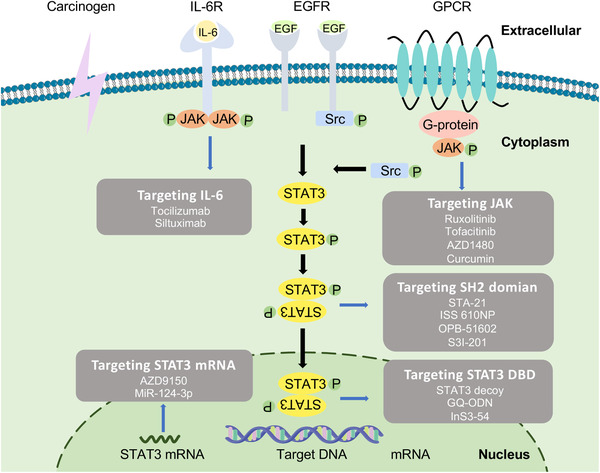
Schematic of STAT3 signaling inhibitors. The principle of STAT3 inhibitors in tumor treatment is based on targeting upstream proteins of the STAT3 signaling or directly targeting STAT3; inhibition of upstream cytokine such as IL‐6 and tyrosine kinases JAK with small‐molecule inhibitors and natural compound such as tocilizumab, siltuximab, curcumin, ruxolitinib. As for inhibiting STAT3 in direct way, NH2‐terminal, DNA‐binding and SH2 domains can be targeted with OPB‐51602, S3I‐201, STAT3 decoy, G‐quartet oligodeoxynucleotide, AZD9150. Negative regulators of STAT3 can also play a role as STAT3 inhibitors (Figure 3)

Considering that aberrantly activated STAT3 is characteristic of cancer formation, progression, and metastasis, recent studies highlighted the effectiveness of blocking STAT3 signaling in cancer treatment (Table 1). In general, constitutive activation of STAT3 signaling can be disrupted by the following mechanisms: (1) indirect STAT3 inhibition by targeting upstream molecules, including IL‐6, EGFR, and JAK; (2) direct STAT3 inhibition by blocking dimerization, preventing target gene transcription, or decreasing total STAT3 expression (Figure 6).

**TABLE 1:** Inhibitors targeting signal transducer and activator of transcription 3 (STAT3) in cancers

**Inhibitor**	**Target**	**Type**	**Cancer type**	**Clinical trials**
Inhibitors targeting upstream receptors				
Dasatinib^203^, ^204^, ^205^, ^206^, ^207^, ^208^	Src	Small molecule	Breast cancer, head and neck squamous cell carcinoma (HNSCC), leukemia, non‐small‐cell lung carcinoma (NSCLC), prostate cancer	NCT00924352 NCT00826449 NCT02744768 NCT00385580
Cetuximab^209^, ^210^, ^211^, ^212^, ^213^	Epidermal growth factor receptor	Antibody	Colorectal cancer, HNSCC	NCT02928224 NCT02164916 NCT02358031
AZD1480^214^, ^215^	Janus kinase (JAK)	Small molecule	HNSCC, colorectal cancer	‐
Ruxolitinib^216^, ^217^, ^218^	JAK	Small molecule	Breast cancer, pancreatic cancer, NSCLC	NCT01423604 NCT01594216 NCT02155465
Tofacitinib^219^	JAK	Small molecule	Lymphocytic leukemia	‐
8αTGH	JAK	Natural compound	HNSCC	‐
Curcumin^221^, ^222^	JAK	Natural compound	Lung cancer, HNSCC, breast cancer	NCT01160302 NCT01740323
WP1066^223^, ^224^, ^225^	JAK	Small molecule	HNSCC, gastric cancer, melanoma	‐
Tocilizumab^226^, ^227^, ^228^	IL‐6R	Antibody	HNSCC, breast cancer, lymphocytic leukemia	NCT03135171 NCT02906371
Siltuximab^229^, ^230^	IL‐6R	Antibody	Prostate cancer, NSCLC	NCT00433446 NCT00841191
Bazedoxifene^231^	IL‐6R	Small molecule	Pancreatic cancer	‐
Metformin^232^, ^233^	IL‐6R	Small molecule	HNSCC, ovarian cancer, breast cancer	NCT01579812 NCT01340300
Inhibitors blocking STAT3 dimerization				
S3I‐M2001^234^	SH2	Peptidomimetic	Breast cancer	‐
S3I‐201^235^, ^236^, ^237^	SH2	Small molecule	AdCC, HNSCC, ASCC	‐
STA‐21^238^	SH2	Small molecule	Breast cancer	‐
Stattic^239^	SH2	Small molecule	Nasopharyngeal carcinoma	‐
OPB‐51602^240^, ^241^, ^242^	SH2	Small molecule	Hematological malignancie, refractory solid malignancies	NCT01344876 NCT01184807
OPB‐111077^243^	SH2	Small molecule	Hepatocellular carcinoma (HCC), acute myeloid leukemia	NCT01711034 NCT03197714
TTI‐101^244^, ^245^	SH2	Small molecule	HCC, HNSCC	‐
CJ‐1383^246^	SH2	Small molecule	Breast cancer	‐
Inhibitors targeting STAT3 DNA‐binding domain (DBD)				
STAT3 decoy^247^, ^248^	DBD	Oligodeoxynucleotides (ODNs)	HNSCC, NSCLC	NCT00696176
G‐quartet ODN^249^, ^250^	DBD	ODNs	HNSCC, NSCLC	‐
InS3‐54A18^251^	DBD	Small molecule	Lung cancer, breast cancer	‐
InS3‐54^252^	DBD	Small molecule	NSCLC, breast cancer	‐
BBI608^253^, ^254^, ^255^	DBD	Small molecule	Colorectal cancer, gastric cancer, glioblastoma	NCT01830621 NCT02315534 NCT02178956
MMPP^256^, ^257^	DBD	Small molecule	NSCLC, ovarian cancer	‐
Inhibitors decreasing STAT3 expression				
AZD9150^258^, ^259^	STAT3 mRNA	Antisense oligonucleotides	Lymphoma, lung cancer, HCC HNSCC	NCT01839604 NCT01563302 NCT03394144 NCT02549651
MiR‐124‐3p^260^	STAT3 mRNA	MicroRNA	Nasopharyngeal carcinoma	‐
SD‐36^261^	STAT3 protein	Small molecule	Lymphocytic leukemia	‐

### 6.1 Targeting the STAT3 pathway for cancer treatment

#### 6.1.1 Targeting upstream receptors

##### Targeting JAK

JAKs are the key activator of the STAT3 pathway. The JAK/STAT pathway is activated by various cytokines, and many JAK inhibitors focus on the potential treatment of chronic inflammatory disorders and CRS.^262^, ^263^, ^264^ The efficacy and safety of several JAK inhibitors are evaluated in clinical trials. Additionally, some orally available, ATP‐competitive, small‐molecule JAK inhibitors are considered for treating solid tumors.^265^ 

Lestaurtinib (CEP‐701), the “first generation” FMS‐like tyrosine kinase‐3 (FLT3) inhibitor that also inhibits JAK2, is reported to inhibit the growth and migration of cancer cells and prevent colony formation of various cancer, including anaplastic thyroid cancer, human neuroblastomas, and mutated acute myeloid leukemia (AML).^266^, ^267^, ^268^ However, Knapper et al. conducted a prospective randomized assessment to evaluate the efficacy of lestaurtinib administered after each cycle of chemotherapy in FLT3‐AML treatment. The results showed that, compared to chemotherapy alone, the combination therapy of lestaurtinib and chemotherapy with concomitant anti‐fungal prophylaxis promoted survival, while no other statistically significant clinical benefit was reported, indicating that lestaurtinib may not be the optimal drug for further AML clinical trials.^269^

Sen et al. showed that AZD1480 abrogated STAT3 phosphorylation induced by IL‐6 and exhibited anti‐tumor activity in HPV‐HNSCC in vivo mouse model (in two patient‐derived HNSCC xenograft models, the tumor volume derived from Patient 1 in the AZD1480 administration group was less than 1/2 compared with the vehicle treatment group).^214^ Although AZD1480 is demonstrated to have a potential anti‐cancer effect, its adverse events in clinical administration remain a problem. In a Phase 1 clinical trial, dose‐limiting toxicities were observed after the administration of AZD1480 in patients with solid tumors.^270^

As a small‐molecule inhibitor of JAK2 phosphorylation, WP1066 shows highly anti‐tumor effectiveness in the treatment of AML, melanoma, and bladder cancer. Besides, WP1066 could improve the chemo‐sensitivity in various tumor models.^271^, ^272^, ^273^ For instance, WP1066 sensitizes OSCC cells to cisplatin by targeting the STAT3/miR‐21 axis. Zhang et al. reported that after the combination of WP1066 and cisplatin in the treatment of Tca8113/DDP xenograft tumor models, the growth rate was significantly less than the untreated group.^223^ Liu et al. demonstrated that AG490 plays a suppressing role in angiogenesis and reducing MDSCs in TME of HNSCC by inhibiting the JAK2/STAT3 pathway.^274^

Ruxolitinib (INC424) is an FDA‐approved drug that targets JAK1/2 to treat inflammatory diseases. Although it is not approved for cancer treatment by FDA, clinical trials have been conducted. Dao et al. conducted a Phase II study of ruxolitinib to test its safety and efficacy in the treatment of chronic neutrophilic leukemia and atypical chronic myeloid leukemia. It was well‐tolerated, with an overall hematologic response rate of 32% among treated patients.^275^

Natural compounds have also been reported to suppress JAK/STAT3 pathway.^276^ Chen et al. introduced a betulinic acid derivative, SH479, which can target the JAK/STAT3 pathway to inhibit arthritis.^277^ Lee et al. demonstrated the function of arctiin in abrogating JAK and Src activation as well as inhibiting the constitutive activation of pSTAT3.^278^ Pouyfung et al. showed that 8α‐tigloyloxyhirsutinolide‐13‐O‐acetate (8αTGH), a compound from *Vernonia cinerea*, has anti‐tumor activity in an HNSCC mouse model through inhibiting the JAK/STAT3 pathway.^220^ JAK inhibitors were mainly designed for autoimmune dysfunction treatment. Although some of them demonstrated anti‐tumor utility, the results of clinical trials for cancer treatment were still modest. Further studies using JAK inhibitors in combination therapies may provide more promising results.

##### Targeting IL‐6 and IL‐6R

As mentioned, IL‐6 is an inflammatory cytokine that activates the JAK1 and JAK2 pathways, leading to STAT3 phosphorylation. IL‐6 is elevated in the serum of cancer patients and has diagnostic and/or prognostic significance.^64^, ^65^ The blockade of IL‐6 activity by IL‐6 and IL‐6R neutralizing antibodies is already used to treat rheumatoid arthritis (RA).^279^ Moreover, it was reported that tocilizumab, an IL‐6R blocking antibody that is FDA‐approved for RA treatment, can sensitize tumor cells to radiation and overcome erlotinib resistance. Tocilizumab in combination with erlotinib is used to treat SQ20B xenograft tumor models. After 40 days of combination therapy, the survival rate of mice was 40%, compared with 0%, in the erlotinib only group, which suggests that this combinatorial approach may improve the treatment response and survival rate of patients.^226^, ^280^

Siltuximab (CNTO‐328) is a monoclonal antibody that can specifically target IL‐6 and prevent its binding to IL‐6R. IL‐6 is upregulated in cholangiocarcinoma and non‐small cell lung cancer, and treatment with siltuximab alters the IL‐6/STAT3 pathway and suppresses the growth and invasion of tumor.^281^, ^282^ The results of Phase I/II and II clinical trials conducted by Angevin et al. and Coward et al., respectively, indicated that siltuximab was safe in ovarian and Kirsten rat sarcoma‐2‐mutant cancers treatment.^283^, ^284^ However, these several clinical trials of siltuximab in treating patients with cancer was still in their early stage such as Phases I or II due to the adverse events during the cancer treatment. For instance, in a Phase II trial that evaluated mitoxantrone/prednisone with or without siltuximab in prostate cancer treatment, the combination group presented higher percentages of patients with severe adverse events, grade P3 adverse events. Three patients in the combination‐treated group were dead due to siltuximab side effects.^285^ Although siltuximab was regarded as a promising drug in cancer treatment, the way to reduce adverse events associated with siltuximab still need to be explored.

#### 6.1.2 Targeting STAT3 directly

##### Blocking dimerization

SH2 domains recognize and bind to select phosphotyrosine residues on receptors and other proteins to form the multiprotein complexes and mediate the intracellular protein–protein interactions.^286^ There are two ways to inhibit STAT3 directly by targeting SH2: blocking the recruitment of activated RTKs or non‐receptor kinases that phosphorylate Tyr705 of STAT3 to the plasma membrane and blocking the dimerization of two activated STAT3 molecules. Two types of inhibitors, including peptides and small molecules targeting the SH2 domain of STAT3, have been reported to reduce the tumor cells proliferation.^286^, ^287^

Peptidomimetic inhibitors, which are designed via amino acid residues in a STAT3 structure‐based manner, can directly interact with the SH2 domain. PY*LKTK can form inactive STAT3:PY*LKTK complexes and effectively reduces levels of active STAT3:STAT3 dimers in vivo.^33^ ISS 610, which is based on PY*L, was reported to induce the apoptosis of STAT3‐dependent transformed cancer cells without affecting normal cells. S3I‐M2001, an oxazole‐based peptidomimetic, can inhibit the growth of tumors in mice with breast cancer.^234^, ^288^ Ac‐pTyr‐Leu‐Pro‐Gln‐Thr‐Val‐NH2, BP‐PM6, BP‐PM7, and PMM‐172 are all STAT3 inhibitors that target the SH2 domain to reduce constitutive STAT3 phosphorylation in cancer cells such as HNSCC and breast cancer cells.^289^, ^290^, ^291^

Non‐peptidic small‐molecule inhibitors also block STAT3 activation. Genini et al. investigated the mechanism by which OPB‐51602 interferes with STAT3 function in cancer cells. Binding of OPB‐51602 to the SH2 domain disrupts intradomain interactions and promotes STAT3 aggregation, and this causes mitochondrial dysfunction and ultimately leads to cancer cell death.^240^ Kim et al. assessed another inhibitor targeting the SH2 domain of STAT3 called ODZ10117. It can inhibit tyrosine phosphorylation and STAT3 dimerization and thereby reduce tumor growth and lung metastasis in patients with breast cancer.^292^ Song et al. identified STA‐21 as the best‐match small‐molecule inhibitor targeting the SH2 domain of STAT3 among nearly 429,000 compounds selected through serial functional evaluation based on their established cell‐based assays. The breast cancer‐bearing mouse was used to evaluate its anti‐tumor effect, and STAT3 phosphorylation and dimerization were presented to be abrogated.^238^ Moreover, studies have shown that STA‐21 treatment can inhibit cell growth, induce apoptosis in bladder cancer cell lines, and improve psoriasis lesions.^293^, ^294^ Schust et al. introduced a small‐molecule inhibitor of STAT3 activation and dimerization called Stattic, which also targets the SH2 domain. Stattic treatment with 10 μM resulted in the apoptosis of approximately 18% of STAT3‐dependent MDA‐MB‐435S breast cancer cells.^34^ Chen et al. reported a novel small‐molecule inhibitor, N4, which can bind to the SH2 of STAT3 and abolish p‐STAT3 (Tyr705). N4 suppresses tumor growth and metastasis in animal models of pancreatic cancer. After 20 days of treatment with 20 μM/kg N4 in pancreatic cancer‐bearing mice, the tumor weights of the mice were significantly lower than those of the control group (*p* < 0.0001, *n* = 6).^295^ Wang et al. reported that HJC0152, designed as an O‐alkylamino‐tethered derivative of niclosamide, can inhibit p‐STAT3 (Tyr705) and decrease the invasion and migration ability of HNSCC cells.^296^ LY5 was designed as a small‐molecule inhibitor that blocks the phosphotyrosine‐binding site of the SH2 domain at nanomolar concentrations. However, Yu et al. showed that the anti‐tumor effects of LY5 were not due to the inhibition of STAT3 phosphorylation.^297^ Bu et al. found that S3I‐201 reduced MDSCs and improved CSC eradication, which enhanced the efficacy of conventional chemotherapy in an HNSCC mouse model. Six weeks after treatment with S3I‐201 in this mouse model, tumor growth was significantly reduced, compared to that in the PBS control group (*p* < 0.001, *n* = 6). Further research has shown that S3I‐201 can inhibit STAT3 activation and thereby impair cancer cell proliferation and immunosuppression without appreciable side effects in a mouse model of HPV‐negative anal squamous cell carcinoma.^236^, ^237^, ^298^

##### *Targeting the STAT3* DBD

As mentioned previously, the function of activated STAT3 in cancer cell proliferation, migration, and invasion relies on the physical interaction between the DBD and DNA after translocation into the nucleus. Inhibitors targeting the STAT3 DBD may also decrease STAT3 activity by disrupting its binding to the target DNA.^299^ Previously, the DBD of STAT3 was considered undruggable because of its potentially limited selectivity.^300^ Turkson et al. reported that platinum‐containing compounds can selectively block STAT3, and it has been shown that platinum (IV)‐containing complexes, CPA‐1 and CPA‐7, disrupt the DNA‐binding capacity of STAT3 and induce tumor regression.^301^ Recently, a novel small‐molecule lead compound called (E)‐2‐methoxy‐4‐(3‐(4‐methoxyphenyl)prop‐1‐en‐1‐yl)phenol (MMPP) was synthesized by Son et al., and it can suppress cancer growth by directly interacting with the DBD of STAT3.^256^ Compared to the bioactive compound (E)‐2,4‐bis(p‐hydroxyphenyl)‐2‐butenal (BHPB), which was synthesized previously, MMPP circumvented the problem caused by the presence of α, β‐unsaturated carbonyl groups in BHPB that form a non‐selective covalent bond, which is conducive to side effects.^256^, ^302^ Leong et al. showed that the using of a STAT3 decoy is an alternative approach for targeting the activated STAT3. Treatment of HNSCC cells with the STAT3 decoy suppresses cancer cells growth and STAT3‐mediated gene expression.^247^ Moreover, the decoy can also block STAT3 activation in vivo, induce apoptosis, and decrease the proliferation of HNSCC cells. After treatment with the STAT3 decoy combined with cisplatin for 10 days in an HNSCC xenograft model, there was a 7.4‐fold increase in the number of apoptotic cells compared to that in the control group (p = 0.002).^303^ Jing et al. developed a series of G‐quartet oligodeoxynucleotides (GQ‐ODNs) as STAT3 inhibitors that can block its DNA‐binding activity. Treatment with GQ‐ODN plus paclitaxel in nude mice with HNSCC tumors over 21 days showed potent efficacy. The mean tumor size decreased by 35%, compared to the 9.4‐fold increase in the untreated control group.^250^

##### Decreasing STAT3 expression

Antisense oligonucleotides (ASOs) can selectively inhibit the translation of STAT3 mRNA by binding to single‐stranded RNA sequences in a complementary fashion.^304^, ^305^ Li et al. showed that treatment with a specific 2‐O‐methoxyethyl‐modified ASOs, used to knock down STAT3 expression in HCC cells can decrease circulating VEGF, reduce neovascularization, and inhibit metastasis and growth of cancer cells.^306^ Oweida et al. demonstrated that treatment with STAT3 ASOs in combination with radiotherapy can increase the anti‐tumor effect and decrease radioresistance. After treatment for LY2 and MOC2 tumor‐bearing mice with STAT3 ASOs and radiation, the average tumor volumes were 53.0 (5.6) mm^3^ and 254.8 (81.6) mm,^3^ respectively, compared to 277.4 (53.8) mm^3^ and 1042.9 (326.8) mm^3^ in mice treated with STAT3 ASOs alone.^307^ AZD9150, a second‐generation ASO, inhibited STAT3 expression and downstream signaling targets by reducing STAT3 mRNA in lymphoma and lung cancer models.^258^ Treatment with AZD9150 combined with cisplatin in mice can significantly sensitize tumors to cisplatin and improve the survival rate, compared to treatment with either agent alone, which demonstrates a potential clinical benefit to overcome chemoresistance in HNSCC.^259^ Moreover, in addition to ASOs, miR‐124‐3p was reported to downregulate the transcription of STAT3 by interfering with its 3′untranslated region, and this was associated with apoptosis of NPC cells as well as inhibition of proliferation, migration, and invasion.^260^

### 6.2 STAT3 pathway inhibitors in clinical trials against cancer

To date, several STAT3 pathway inhibitors are approved by the FDA for clinical cancer treatment. However, various other STAT3 pathway inhibitors have been used in clinical trials. As mentioned above, lestaurtinib (NCT00557193), AZD1480, ruxolitinib (NCT02092324), and siltuximab have been evaluated for their safety and anti‐tumor effects in cancer treatment in clinical trials.^269^, ^270^, ^275^, ^283^, ^308^, ^309^ EGFR, an activator of the STAT3 pathway, is targeted in clinical trials. Cetuximab is a monoclonal antibody against EGFR that can inhibit STAT3 activation. Carter et al. conducted a Phase 1 trial (NCT01445405) to explore the effect of bortezomib and cetuximab in combination with radiation therapy for advanced HNSCC. The median progression‐free survival of patients treated with combination therapies was just 4.8 months, which indicated limited anti‐tumor efficacy.^310^ However, a Phase 2 trial (NCT00084318) showed that delivery of postoperative chemoradiotherapy (using cisplatin or docetaxel) with cetuximab for HNSCC treatment achieved 69% and 79% 2‐year overall survival and 57% and 66% 2‐year disease‐free survival.^311^

Besides the inhibitors of STAT3 pathway activators, including JAK, IL‐6, and EGFR, the results of drugs directly targeting STAT3 in early‐phase clinical trials demonstrated their potential in clinical use. Grandis et al. conducted an early Phase 0 trial (NCT00696176) of a STAT3 decoy for HNSCC treatment, and results demonstrated that none of the patients suffered Grade 3/4 or dose‐limiting toxicities. Moreover, patients with HNSCC received intratumoral injections of the STAT3 decoy before surgery, and levels of STAT3 target genes, including cyclin D1 and Bcl‐XL, were reduced, compared to those in the control group (saline).^312^ Owing to the promising anti‐tumor effect of AZD9150 in preclinical studies, Hong et al. conducted a Phase I dose‐escalation clinical trial. A total of 25 patients with lymphoma and lung cancer was included in this study; 5% of them experienced drug‐related adverse events, and 11 of the 25 evaluated patients had stable disease or partial response. Moreover, three of six patients had refractory diffuse large B‐cell lymphoma (DLBCL), and two presented with a durable partial response.^258^ Subsequently, AZD9150 was demonstrated to be well‐tolerated, and four of 30 patients with DLBCL achieved a response for at least 4 months associated with a 13% of clinical benefit rate in a Phase 1b clinical trial (NCT01563302).^313^ In 2016, BBI608, a small molecule that directly targets the STAT3 DBD to inhibit STAT3 activation, was approved by the FDA for GEJ and pancreatic cancer treatment. Later, Jonker et al. conducted a Phase III clinical trial (NCT01830621) to test the efficacy of BBI608 for the treatment of refractory advanced colorectal cancer, and the results showed that BBI608 can prolong overall survival to 5.1 months in patients with pSTAT3‐positive tumors, compared to 3 months in the placebo group.^253^

### 6.3 Drug delivery systems for STAT3 inhibitors

In cancer therapy, STAT3 inhibitors play a significant role in improving anti‐tumor efficacy and can augment conventional chemotherapeutic effects.^314^, ^315^ However, conventional STAT3 inhibitors have some limitations, including the lack of anti‐tumor effects, low selectivity, and high toxicity. To overcome these barriers, many studies have introduced novel drug delivery systems for STAT3 inhibitors to overcome these limitations. In nanomaterial‐based drug delivery systems, STAT3 inhibitors described above are encapsulated into nanomaterials including biomimetic materials, liposomes, polymers, and inorganic materials (Table 2). Compared to the conventional STAT3 inhibitors, nanomaterial‐based drug delivery systems have the following advantages: (1) biocompatibility and tumor targeting that reduce cytotoxicity to normal tissues; (2) efficient drug‐loading capacity that can be used as a carrier for more than one drug; and (3) protection of loaded drugs from clearance during blood circulation.^316^, ^317^

**TABLE 2:** Drug delivery system of STAT3 inhibitors in cancer treatment

**Material**	**Drug**	**Delivery routes**	**Cells/Tissues Specificity**	**Ref**
Liposomes	Curcumin‐loaded liposomes‐STAT3 siRNA	Intratumoral administration	Skin cancer	^318^
	Hyaluronic acid/TN‐CCLP	Intravenous administration	Breast cancer	^319^
	Rop‐DPRL and calorie restriction	Intraperitoneal injection	Melanoma	^320^
	Stattic	–	Melanoma cells	^321^
Polymers	Gel‐ NSC74859‐ICG	Intravenous administration	HNSCC	^322^
	Ritonavir derivative	Intravenous administration	HNSCC	^323^
	Cucurbitacin‐D; doxorubicin	Intravenous administration	Breast cancer	^324^
	HA/^siSTAT3^PPL_PTX_	Intravenous administration	Breast cancer	^325^
Biomimetic material	Exo‐JSI124	Intranasal delivery	Glioblastoma tumor	^326^
	Tumor‐derived exosomes‐ miR‐34a	–	Colorectal cancer cells	^327^
	Corosolic acid‐long‐circulating liposomes‐αCD163	–	Tumor‐associated macrophages	^328^
	NPs‐αIL6R Ab‐CD44	Intravenous administration	Breast cancer	^329^
	CaP‐cored low‐density lipoprotein nanovehicle‐STAT3 decoy ODNs	Intravenous administration	HCC cells	^330^
Inorganic material	AuNP‐NUAP‐STAT3d	–	HNSCC cells	^331^
	AIRISE‐02(STAT3 siRNA‐CpG‐mesoporous silica nanoparticle)	Intratumoral administration	Breast cancer	^332^
	Layer‐by‐layer assembled gold nanoparticles‐STAT3 siRNA ‐imatinib	Intratumoral administration	Melanoma	^333^
	SPION‐TMC‐ChT‐TAT‐H NPs	Intravenous administration	Colorectal cancer	^334^

Liposomes, which are cell membrane‐like structures, have an efficient drug‐loading ability as well as biocompatibility; hydrophobic drugs can be readily loaded inside the lipid bilayer membranes.^335^ Shi et al. designed a calcium phosphate‐cored low‐density lipoprotein nanovehicle as a Trojan horse to load STAT3 decoy ODNs to overcome tumor necrosis factor‐related apoptosis‐inducing ligand resistance.^330^ Wu et al. introduced PEGylated liposomal FLLL32, a specific STAT3 inhibitor that binds to the SH2 domain. It enhanced anti‐tumor efficacy and reduced toxicity through intravenous administration in treating pancreatic cancer.^336^ Exosomes that are liposomes with antigens, mRNAs, and miRNAs derived from various cells are promising vehicles for cancer treatment to overcome the tumor barrier to drug delivery. Zhuang et al. introduced a non‐invasive method to deliver a STAT3 inhibitor loaded into exosomes via an intranasal route. This inhibitor can be taken up by microglial cells, and it can prolong the survival time of GL26 tumor‐bearing mice to 44.5 days, compared to the control group.^326^

Zheng et al. introduced poly lactic‐co‐glycolic acid (PLGA) nanoparticles to co‐deliver the cancer drug doxorubicin (Dox) and the STAT3 inhibitor nifurate (DNNPs) and achieve long‐term drug release. After 2 h of treatment with DNNPs, the fluorescence intensity of Dox, which reflected DNNP uptake by BGC‐823 gastric tumors in mice, was approximately three‐fold that of the Dox control group (*p* < 0.001).^337^ Tavares et al. introduced HPMA‐based copolymers containing cucurbitacin‐D (CuD), a STAT3 inhibitor, and Dox. In this drug delivery system, CuD can be released in a sustained, controlled manner and can effectively target breast cancer. After treatment with linear‐CuD in combination with micellar‐Dox in 4T1 tumor‐bearing mice, the tumor volume decreased significantly, compared to that in the linear‐CuD control group.^324^ Additionally, drug delivery systems introduced by Lavasanifar and colleagues including PEO‐b‐P(CL‐JSI‐124) conjugates, the PLGA‐JSI‐124 conjugate, PEO‐b‐PBCL micelles are demonstrated to be efficacy in cancer treatment.^338^, ^339^, ^340^

In addition to enhancing the anti‐tumor efficacy of STAT3 inhibitors through different drug delivery systems. STAT3 inhibitors can also be combined with other therapies to enhance the efficacy and safety of tumor treatment strategies. The immune checkpoint protein blockade is a promising immunotherapy for cancer treatment. However, resistance to durvalumab (the anti‐PD‐L1 antibody) and tremelimumab (the anti‐CTLA4 antibody) associated with STK11 gene mutations is reported in non‐small‐cell lung carcinoma (NSCLC) treatment, and this has impacted the efficacy of immune blockade. It was demonstrated that STAT3 ASOs reversed this resistance, and STAT3 ASOs administration in combination with immune checkpoint blockade significantly enhanced the anti‐tumor efficacy, compared to immunotherapy alone.^341^

CAR‐T cell therapy is a novel immunotherapy that demonstrated excellent efficacy in the treatment of hematological malignancies. However, overexpression of cytokines, such as IL‐6 and IL‐10, caused by STAT3 hyperactivation is associated with the occurrence of CRS. Therefore, treatment with the STAT3 signaling pathway inhibitor tocilizumab in combination with CAR‐T cell therapy can reduce adverse events without impeding CAR‐T cell expansion.^342^ Guha et al. demonstrated that STAT3 inhibitors of liver‐associated MDSCs (STATTIC or BBI608) can enhance the anti‐tumor efficacy of CAR‐T cells. In the murine liver metastasis model, STAT3 inhibition did not decrease CAR‐T cell cytotoxicity nor cause the death of CAR‐T cells, and apoptosis of tumor cells dose‐dependently increased, compared to the control group.^343^ Because STAT3 is associated with upregulation of PD‐L1 expression, STAT3 inhibitors play a role in immunotherapy, and STAT3 inhibition combined with photothermal therapy based on gelatinase‐sensitive gelatin nanoparticles was reported to enhance anti‐tumor efficacy in HNSCC cancer treatment.^133^ On the 15th day after combination therapy, levels of immunosuppressive MDSCs and PD‐1 in the blood, tumor, and spleen were significantly reduced, and the growth of tumor volume was limited in the *Tgfbr1/Pten* double conditional knockout (2cKO) mice model, compared to the untreated group.^322^

As mentioned, STAT3 inhibitors loaded into organic materials, inorganic materials, and unique biomimetic materials have demonstrated great tumor‐targeting, biocompatibility, and anti‐tumor efficacy.

## 7 CONCLUSION AND PERSPECTIVES

The STAT3 pathway plays a physiological role in normal cell growth, differentiation, and survival. Meanwhile, as the “Achilles’ heel” of many cancers, owing to the association of constitutive STAT3 activation with cancer hallmarks as well as the relationship between STAT3 pathway and poor patient outcomes, many researchers believe that STAT3 acts as an oncogene and that blockade of STAT3 activity is a viable strategy for cancer treatment. Additionally, based on clinical trial results, various STAT3 inhibitors are well‐tolerated and potentially improve the overall survival of patients with cancer.

Despite improvements in STAT3 targeting therapeutics, the conventional view is that STAT3 is “undruggable” owing to its ubiquitous expression. Only a few clinically available STAT3 inhibitors for cancer treatment are approved by the FDA, and there are several concerns that remain worthy of attention and consideration. First, STAT3 inhibitors should not affect the functions of structurally similar proteins, such as STAT1, and the side effects of STAT3 inhibitors, including hematologic toxicity, are still major problems to be addressed. Further efforts should be made to clarify the mechanisms of selectivity for STAT3 inhibition and to explore the novel delivery of STAT3 inhibitors to reduce toxicity, increase local concentrations, enhance cellular internalization, and achieve long‐term release. Second, the development of STAT3 inhibitors is focused on STAT3:STAT3 dimerization, which is a dilemma because of the lack of a reliable off‐target biomarker. Thus, in addition to the SH2 domain, further research should explore the targeting of other STAT3 domains. Recently, with the development of purchasable chemical databases and computer‐aided drug discovery, the undruggable STAT3–DNA interface is targeted via artificial intelligence, which can virtually screen (VS) billions of molecular structures.^344^ For example, inS3‐54, an inhibitor of STAT3 that targets its DBD, was designed via a VS of 200,000 compounds that excluded molecules predicted to bind STAT1. Moreover, to prevent off‐target side effects, inS3‐54 analogs were searched in the virtual Chemdiv database, and analog A18 was demonstrated to specifically bind to the DBD of STAT3 and inhibit its activity.^251^, ^252^ Third, the drug delivery system and the combination of STAT3 inhibitors with other anti‐tumor therapies can greatly enhance cancer treatment. For instance, STAT3 inhibitors in combination with CAR‐T cell therapy can reduce adverse events, STAT3 inhibitors loaded into nanocarriers have enhanced serum stability to promote anti‐cancer efficacy, and STAT3 inhibitors in combination with radiation can overcome radiotherapy resistance. In summary, with the progress of research on STAT3 pathway and advances in clinical trials of STAT3 inhibitors, the significance of STAT3 pathway in anti‐tumor activity is evident. In the future, multimodal delivery of drugs targeting STAT3 pathway and other conventional therapies could be a promising strategy for cancer treatment.

## CONFLICT OF INTEREST

The authors have no conflict of interest to declare.

## ETHICS STATEMENT

No ethical approval was required for this study.

## AUTHOR CONTRIBUTION

L.L.B., L.L.C., and B.L. conceived the idea; H.Q.W., Q.W.M., and F.Y.H. performed the literature search and drafted the manuscript; X.G., H.L., S.R.L., J.W., F.C.S., Y.S. revised and edited the manuscript; L.L.B., L.L.C., and B.L. supervised and revised the manuscript. The authors read and approved the final manuscript.

## Data Availability

Not applicable.

## References

[1] Sung H , Ferlay J , Siegel RL , et al. Global cancer statistics 2020: gLOBOCAN estimates of incidence and mortality worldwide for 36 cancers in 185 countries. CA Cancer J Clin. 2021;71(3):209‐249. 33538338 10.3322/caac.21660

[2] World Health Organization . World Health Statistics 2021: Monitoring Health for the SDGs, Sustainable Development Goals. World Health Organization; 2021.

[3] Ciombor KK , Bekaii‐Saab T . A comprehensive review of sequencing and combination strategies of targeted agents in metastatic colorectal cancer. Oncologist. 2018;23(1):25. 29021377 10.1634/theoncologist.2017-0203 PMC5759820

[4] Addeo A , Banna GL , Metro G , Di Maio M . Chemotherapy in combination with immune checkpoint inhibitors for the first‐line treatment of patients with advanced non‐small cell lung cancer: a systematic review and literature‐based meta‐analysis. Front Oncol. 2019;9:264. 31058078 10.3389/fonc.2019.00264 PMC6478036

[5] Chen J , Ni Y , Sun G , et al. Comparison of current systemic combination therapies for metastatic hormone‐sensitive prostate cancer and selection of candidates for optimal treatment: a systematic review and Bayesian network meta‐analysis. Front Oncol. 2020;10:1806. 10.3389/fonc.2020.519388 PMC7531177 33072564

[6] Dobson GP . Addressing the global burden of trauma in major surgery. Front Surg. 2015;2:43‐43. 26389122 10.3389/fsurg.2015.00043 PMC4558465

[7] Nikolaou M , Pavlopoulou A , Georgakilas AG , Kyrodimos E . The challenge of drug resistance in cancer treatment: a current overview. Clin Exp Metastasis. 2018;35(4):309‐318. 29799080 10.1007/s10585-018-9903-0

[8] Nurgali K , Jagoe RT , Abalo R . Adverse effects of cancer chemotherapy: anything new to improve tolerance and reduce sequelae? Front Pharmacol. 2018;9:245. 29623040 10.3389/fphar.2018.00245 PMC5874321

[9] Loh C‐Y , Arya A , Naema AF , Wong WF , Sethi G , Looi CY . Signal transducer and activator of transcription (STATs) proteins in cancer and inflammation: functions and therapeutic implication. Front Oncol. 2019;9:48. 30847297 10.3389/fonc.2019.00048 PMC6393348

[10] Wegrzyn J , Potla R , Chwae YJ , et al. Function of mitochondrial Stat3 in cellular respiration. Science. 2009;323(5915):793‐797. 19131594 10.1126/science.1164551 PMC2758306

[11] Kano A , Wolfgang MJ , Gao Q , et al. Endothelial cells require STAT3 for protection against endotoxin‐induced inflammation. J Exp Med. 2003;198(10):1517‐1525. 14623907 10.1084/jem.20030077 PMC2194113

[12] Laouar Y , Welte T , Fu XY , Flavell RA . STAT3 is required for Flt3L‐dependent dendritic cell differentiation. Immunity. 2003;19(6):903‐912. 14670306 10.1016/s1074-7613(03)00332-7

[13] Moh A , Iwamoto Y , Chai GX , et al. Role of STAT3 in liver regeneration: survival, DNA synthesis, inflammatory reaction and liver mass recovery. Lab Invest. 2007;87(10):1018‐1028. 17660847 10.1038/labinvest.3700630

[14] Hirano T , Ishihara K , Hibi M . Roles of STAT3 in mediating the cell growth, differentiation and survival signals relayed through the IL‐6 family of cytokine receptors. Oncogene. 2000;19(21):2548‐2556. 10851053 10.1038/sj.onc.1203551

[15] Kim DY , Cha ST , Ahn DH , et al. STAT3 expression in gastric cancer indicates a poor prognosis. J Gastroenterol Hepatol. 2009;24(4):646‐651. 19175826 10.1111/j.1440-1746.2008.05671.x

[16] Xu Y , Lu S . A meta‐analysis of STAT3 and phospho‐STAT3 expression and survival of patients with non‐small‐cell lung cancer. Eur J Surg Oncol. 2014;40(3):311‐317. 24332948 10.1016/j.ejso.2013.11.012

[17] Kusaba T , Nakayama T , Yamazumi K , et al. Activation of STAT3 is a marker of poor prognosis in human colorectal cancer. Oncol Rep. 2006;15(6):1445‐1451. 16685378

[18] Zhao Y , Zhang J , Xia H , et al. Stat3 is involved in the motility, metastasis and prognosis in lingual squamous cell carcinoma. Cell Biochem Funct. 2012;30(4):340‐346. 22302289 10.1002/cbf.2810

[19] Johnson DE , Burtness B , Leemans CR , Lui VWY , Bauman JE , Grandis JR . Head and neck squamous cell carcinoma. Nat Rev Dis Primers. 2020;6(1):92. 33243986 10.1038/s41572-020-00224-3 PMC7944998

[20] Huynh J , Chand A , Gough D , Ernst M . Therapeutically exploiting STAT3 activity in cancer‐using tissue repair as a road map. Nat Rev Cancer. 2019;19(2):82‐96. 30578415 10.1038/s41568-018-0090-8

[21] Yu H , Pardoll D , Jove R . STATs in cancer inflammation and immunity: a leading role for STAT3. Nat Rev Cancer. 2009;9(11):798‐809. 19851315 10.1038/nrc2734 PMC4856025

[22] Lee H , Jeong AJ , Ye SK . Highlighted STAT3 as a potential drug target for cancer therapy. BMB Rep. 2019;52(7):415‐423. 31186087 10.5483/BMBRep.2019.52.7.152 PMC6675244

[23] Fu X‐Y , Kessler DS , Veals SA , Levy DE , Darnell J . ISGF3, the transcriptional activator induced by interferon alpha, consists of multiple interacting polypeptide chains. Proc Natl Acad Sci. 1990;87(21):8555‐8559. 2236065 10.1073/pnas.87.21.8555 PMC54995

[24] Fu XY . A transcription factor with SH2 and SH3 domains is directly activated by an interferon alpha‐induced cytoplasmic protein tyrosine kinase(s). Cell. 1992;70(2):323‐335. 1638633 10.1016/0092-8674(92)90106-m

[25] Wegenka UM , Buschmann J , Lütticken C , Heinrich PC , Horn F . Acute‐phase response factor, a nuclear factor binding to acute‐phase response elements, is rapidly activated by interleukin‐6 at the posttranslational level. Mol Cell Biol. 1993;13(1):276‐288. 7678052 10.1128/mcb.13.1.276-288.1993 PMC358907

[26] Akira S , Nishio Y , Inoue M , et al. Molecular cloning of APRF, a novel IFN‐stimulated gene factor 3 p91‐related transcription factor involved in the gp130‐mediated signaling pathway. Cell. 1994;77(1):63‐71. 7512451 10.1016/0092-8674(94)90235-6

[27] Sgrignani J , Garofalo M , Matkovic M , Merulla J , Catapano CV , Cavalli A . Structural biology of STAT3 and its implications for anticancer therapies development. Int J Mol Sci. 2018;19(6):1591. 10.3390/ijms19061591 PMC6032208 29843450

[28] Mertens C , Haripal B , Klinge S , Darnell JE . Mutations in the linker domain affect phospho‐STAT3 function and suggest targets for interrupting STAT3 activity. Proc Natl Acad Sci. 2015;112(48):14811‐14816. 26553978 10.1073/pnas.1515876112 PMC4672786

[29] Hu T , Yeh JE , Pinello L , et al. Impact of the N‐terminal domain of STAT3 in STAT3‐dependent transcriptional activity. Mol Cell Biol. 2015;35(19):3284‐3300. 26169829 10.1128/MCB.00060-15 PMC4561728

[30] Zhang T , Kee WH , Seow KT , Fung W , Cao X . The coiled‐coil domain of Stat3 is essential for its SH2 domain‐mediated receptor binding and subsequent activation induced by epidermal growth factor and interleukin‐6. Mol Cell Biol. 2000;20(19):7132‐7139. 10982829 10.1128/mcb.20.19.7132-7139.2000 PMC86266

[31] Haan S , Hemmann U , Hassiepen U , et al. Characterization and binding specificity of the monomeric STAT3‐SH2 domain. J Biol Chem. 1999;274(3):1342‐1348. 9880505 10.1074/jbc.274.3.1342

[32] Bromberg JF , Wrzeszczynska MH , Devgan G , et al. Stat3 as an oncogene. Cell. 1999;98(3):295‐303. 10458605 10.1016/s0092-8674(00)81959-5

[33] Turkson J , Ryan D , Kim JS , et al. Phosphotyrosyl peptides block Stat3‐mediated DNA binding activity, gene regulation, and cell transformation. J Biol Chem. 2001;276(48):45443‐45455. 11579100 10.1074/jbc.M107527200

[34] Schust J , Sperl B , Hollis A , Mayer TU , Berg T . Stattic: a small‐molecule inhibitor of STAT3 activation and dimerization. Chem Biol. 2006;13(11):1235‐1242. 17114005 10.1016/j.chembiol.2006.09.018

[35] Slebos RJ , Kibbelaar RE , Dalesio O , et al. K‐ras oncogene activation as a prognostic marker in adenocarcinoma of the lung. N Engl J Med. 1990;323(9):561‐565. 2199829 10.1056/NEJM199008303230902

[36] Jove R , Hanafusa H . Cell transformation by the viral src oncogene. Annu Rev Cell Biol. 1987;3(1):31‐56. 2446642 10.1146/annurev.cb.03.110187.000335

[37] Watson CJ , Miller WR . Elevated levels of members of the STAT family of transcription factors in breast carcinoma nuclear extracts. Br J Cancer. 1995;71(4):840‐844. 7710952 10.1038/bjc.1995.162 PMC2033751

[38] Levy DE , Inghirami G . STAT3: a multifaceted oncogene. Proc Natl Acad Sci U S A. 2006;103(27):10151‐10152. 16801534 10.1073/pnas.0604042103 PMC1502425

[39] Attili I , Karachaliou N , Bonanno L , et al. STAT3 as a potential immunotherapy biomarker in oncogene‐addicted non‐small cell lung cancer. Ther Adv Med Oncol. 2018;10:1758835918763744. 29636826 10.1177/1758835918763744 PMC5888808

[40] Resemann HK , Watson CJ , Lloyd‐Lewis B . The Stat3 paradox: a killer and an oncogene. Mol Cell Endocrinol. 2014;382(1):603‐611. 23827176 10.1016/j.mce.2013.06.029

[41] Tolomeo M , Cascio A . The multifaced role of STAT3 in cancer and its implication for anticancer therapy. Int J Mol Sci. 2021;22(2):603. 10.3390/ijms22020603 PMC7826746 33435349

[42] Grandis JR , Drenning SD , Zeng Q , et al. Constitutive activation of Stat3 signaling abrogates apoptosis in squamous cell carcinogenesis in vivo. Proc Natl Acad Sci U S A. 2000;97(8):4227‐4232. 10760290 10.1073/pnas.97.8.4227 PMC18206

[43] Lin GS , Yang LJ , Wang XF , et al. STAT3 Tyr705 phosphorylation affects clinical outcome in patients with newly diagnosed supratentorial glioblastoma. Med Oncol. 2014;31(4):924. 24652192 10.1007/s12032-014-0924-5

[44] Fei Y , Yu J , Li Y , et al. Plasma soluble PD‐L1 and STAT3 predict the prognosis in diffuse large B cell lymphoma patients. J Cancer. 2020;11(23):7001. 33123290 10.7150/jca.47816 PMC7591999

[45] Song JI , Grandis JR . STAT signaling in head and neck cancer. Oncogene. 2000;19(21):2489‐2495. 10851047 10.1038/sj.onc.1203483

[46] Trivedi S , Concha‐Benavente F , Srivastava RM , et al. Immune biomarkers of anti‐EGFR monoclonal antibody therapy. Ann Oncol. 2015;26(1):40‐47. 24997207 10.1093/annonc/mdu156 PMC4269339

[47] Lai SY , Johnson FM . Defining the role of the JAK‐STAT pathway in head and neck and thoracic malignancies: implications for future therapeutic approaches. Drug Resist Updat. 2010;13(3):67‐78. 20471303 10.1016/j.drup.2010.04.001

[48] Luo J , Wang K , Yeh S , et al. LncRNA‐p21 alters the antiandrogen enzalutamide‐induced prostate cancer neuroendocrine differentiation via modulating the EZH2/STAT3 signaling. Nat Commun. 2019;10(1):2571. 31189930 10.1038/s41467-019-09784-9 PMC6561926

[49] Li X , Gera L , Zhang S , et al. Pharmacological inhibition of noncanonical EED‐EZH2 signaling overcomes chemoresistance in prostate cancer. Theranostics. 2021;11(14):6873‐6890. 34093859 10.7150/thno.49235 PMC8171087

[50] Shinagawa K , Yanamoto S , Naruse T , et al. Clinical roles of interleukin‐6 and STAT3 in oral squamous cell carcinoma. Pathol Oncol Res. 2017;23(2):425‐431. 27744625 10.1007/s12253-016-0134-x

[51] Pectasides E , Egloff AM , Sasaki C , et al. Nuclear localization of signal transducer and activator of transcription 3 in head and neck squamous cell carcinoma is associated with a better prognosis. Clin Cancer Res. 2010;16(8):2427‐2434. 20371693 10.1158/1078-0432.CCR-09-2658 PMC3030188

[52] Dolled‐Filhart M , Camp RL , Kowalski DP , Smith BL , Rimm DL . Tissue microarray analysis of signal transducers and activators of transcription 3 (Stat3) and phospho‐Stat3 (Tyr705) in node‐negative breast cancer shows nuclear localization is associated with a better prognosis. Clin Cancer Res. 2003;9(2):594‐600. 12576423

[53] Sonnenblick A , Shriki A , Galun E , et al. Tissue microarray‐based study of patients with lymph node‐positive breast cancer shows tyrosine phosphorylation of signal transducer and activator of transcription 3 (tyrosine705‐STAT3) is a marker of good prognosis. Clin Transl Oncol. 2012;14(3):232‐236. 22374428 10.1007/s12094-012-0789-z

[54] Pencik J , Schlederer M , Gruber W , et al. STAT3 regulated ARF expression suppresses prostate cancer metastasis. Nat Commun. 2015;6:7736. 26198641 10.1038/ncomms8736 PMC4525303

[55] Schindler C . STATs as activators of apoptosis. Trends Cell Biol. 1998;8(3):97‐98. 9695817 10.1016/s0962-8924(98)01233-1

[56] Bromberg J . The role of STATs in transcriptional control and their impact on cellular function. Oncogene. 2000;19(21):2468‐2473. 10851045 10.1038/sj.onc.1203476

[57] Mui AL . The role of STATs in proliferation, differentiation, and apoptosis. Cell Mol Life Sci. 1999;55(12):1547‐1558. 10526572 10.1007/s000180050394 PMC11146798

[58] Zhong Z , Wen Z , Darnell JE . Stat3: a STAT family member activated by tyrosine phosphorylation in response to epidermal growth factor and interleukin‐6. Science. 1994;264(5155):95‐98. 8140422 10.1126/science.8140422

[59] Fan Y , Mao R , Yang J . NF‐kappaB and STAT3 signaling pathways collaboratively link inflammation to cancer. Protein Cell. 2013;4(3):176‐185. 23483479 10.1007/s13238-013-2084-3 PMC4875500

[60] Horvath CM , Wen Z . A STAT protein domain that determines DNA sequence recognition suggests a novel DNA‐binding domain. Genes Dev. 1995;9(8):984‐994. 7774815 10.1101/gad.9.8.984

[61] Hu X , li J , Fu M , Zhao X , Wang W . The JAK/STAT signaling pathway: from bench to clinic. Signal Transduc Target Ther. 2021;6(1):402. 10.1038/s41392-021-00791-1 PMC8617206 34824210

[62] Mali SB . Review of STAT3 (Signal Transducers and Activators of Transcription) in head and neck cancer. Oral Oncol. 2015;51(6):565‐569. 25817923 10.1016/j.oraloncology.2015.03.004

[63] Bournazou E , Bromberg J . Targeting the tumor microenvironment: JAK‐STAT3 signaling. JAKSTAT. 2013;2(2):e23828. 24058812 10.4161/jkst.23828 PMC3710325

[64] Sansone P , Bromberg J . Targeting the interleukin‐6/Jak/stat pathway in human malignancies. J Clin Oncol. 2012;30(9):1005‐1014. 22355058 10.1200/JCO.2010.31.8907 PMC3341105

[65] Dineshkumar T , Ashwini BK , Rameshkumar A , Rajashree P , Ramya R , Rajkumar K . Salivary and serum interleukin‐6 levels in oral premalignant disorders and squamous cell carcinoma: diagnostic value and clinicopathologic correlations. Asian Pac J Cancer Prev. 2016;17(11):4899‐4906. 28032493 10.22034/APJCP.2016.17.11.4899 PMC5454693

[66] Sriuranpong V , Park JI , Amornphimoltham P , Patel V , Nelkin BD , Gutkind JS . Epidermal growth factor receptor‐independent constitutive activation of STAT3 in head and neck squamous cell carcinoma is mediated by the autocrine/paracrine stimulation of the interleukin 6/gp130 cytokine system. Cancer Res. 2003;63(11):2948‐2956. 12782602

[67] Johnson DE , O'Keefe RA , Grandis JR . Targeting the IL‐6/JAK/STAT3 signalling axis in cancer. Nat Rev Clin Oncol. 2018;15(4):234‐248. 29405201 10.1038/nrclinonc.2018.8 PMC5858971

[68] Jin W . Role of JAK/STAT3 signaling in the regulation of metastasis, the transition of cancer stem cells, and chemoresistance of cancer by epithelial‐mesenchymal transition. Cells. 2020;9(1):217. 10.3390/cells9010217 PMC7017057 31952344

[69] Shi T , Papay RS , Perez DM . α(1A)‐adrenergic receptor differentially regulates STAT3 phosphorylation through PKCϵ and PKCδ in myocytes. J Recept Signal Transduct Res. 2012;32(2):76‐86. 22268811 10.3109/10799893.2011.647353 PMC3624730

[70] Chang YF , Liu TY , Liu ST , Tseng CN . Arecoline inhibits myogenic differentiation of C2C12 myoblasts by reducing STAT3 phosphorylation. Food Chem Toxicol. 2012;50(10):3433‐3439. 22847137 10.1016/j.fct.2012.07.032

[71] Fan TF , Deng WW , Bu LL , Wu TF , Zhang WF , Sun ZJ . B7‐H3 regulates migration and invasion in salivary gland adenoid cystic carcinoma via the JAK2/STAT3 signaling pathway. Am J Transl Res. 2017;9(3):1369‐1380. 28386362 PMC5376027

[72] Li P , Huang T , Zou Q , et al. FGFR2 promotes expression of PD‐L1 in colorectal cancer via the JAK/STAT3 signaling pathway. J Immunol. 2019;202(10):3065‐3075. 30979816 10.4049/jimmunol.1801199

[73] Tan Y , Shu L , Xu P , Bai S . Mesenchymal stem cells attract endothelial progenitor cells via a positive feedback loop between CXCR2 and CXCR4. Stem Cells Int. 2019;2019:4197164. 31885605 10.1155/2019/4197164 PMC6915119

[74] Yu CL , Meyer DJ , Campbell GS , et al. Enhanced DNA‐binding activity of a Stat3‐related protein in cells transformed by the Src oncoprotein. Science. 1995;269(5220):81‐83. 7541555 10.1126/science.7541555

[75] Silva CM . Role of STATs as downstream signal transducers in Src family kinase‐mediated tumorigenesis. Oncogene. 2004;23(48):8017‐8023. 15489919 10.1038/sj.onc.1208159

[76] Cirri P , Chiarugi P , Marra F , et al. c‐Src activates both STAT1 and STAT3 in PDGF‐stimulated NIH3T3 cells. Biochem Biophys Res Commun. 1997;239(2):493‐497. 9344858 10.1006/bbrc.1997.7493

[77] Zhao C , Yang L , Zhou F , et al. Feedback activation of EGFR is the main cause for STAT3 inhibition‐irresponsiveness in pancreatic cancer cells. Oncogene. 2020;39(20):3997‐4013. 32242147 10.1038/s41388-020-1271-y

[78] Ashrafizadeh M , Gholami MH , Mirzaei S , et al. Dual relationship between long non‐coding RNAs and STAT3 signaling in different cancers: new insight to proliferation and metastasis. Life Sci. 2021;270:119006. 33421521 10.1016/j.lfs.2020.119006

[79] Dong H , Lei J , Ding L , Wen Y , Ju H , Zhang X . MicroRNA: function, detection, and bioanalysis. Chem Rev. 2013;113(8):6207‐6233. 23697835 10.1021/cr300362f

[80] Kohanbash G , Okada H . MicroRNAs and STAT interplay. Semin Cancer Biol. 2012;22(1):70‐75. 22210182 10.1016/j.semcancer.2011.12.010 PMC3288787

[81] Zhou W , Wang L , Miao Y , Xing R . Novel long noncoding RNA GACAT3 promotes colorectal cancer cell proliferation, invasion, and migration through miR‐149. Onco Targets Ther. 2018;11:1543‐1552. 29593420 10.2147/OTT.S144103 PMC5865577

[82] Liang C , Zhao T , Li H , et al. Long non‐coding RNA ITIH4‐AS1 accelerates the proliferation and metastasis of colorectal cancer by activating JAK/STAT3 signaling. Mol Ther Nucleic Acids. 2019;18:183‐193. 31557619 10.1016/j.omtn.2019.08.009 PMC6796638

[83] Deng S , Wang J , Zhang L , Li J , Jin Y . LncRNA HOTAIR promotes cancer stem‐like cells properties by sponging miR‐34a to activate the JAK2/STAT3 pathway in pancreatic ductal adenocarcinoma. Onco Targets Ther. 2021;14:1883‐1893. 33737813 10.2147/OTT.S286666 PMC7966354

[84] Xia D , Yao R , Zhou P , Wang C , Xia Y , Xu S . LncRNA NEAT1 reversed the hindering effects of miR‐495‐3p/STAT3 axis and miR‐211/PI3K/AKT axis on sepsis‐relevant inflammation. Mol Immunol. 2020;117:168‐179. 31812793 10.1016/j.molimm.2019.10.009

[85] Li R , Chen S , Zhan J , et al. Long noncoding RNA FOXD2‐AS1 enhances chemotherapeutic resistance of laryngeal squamous cell carcinoma via STAT3 activation. Cell Death Dis. 2020;11(1):41. 31959918 10.1038/s41419-020-2232-7 PMC6971019

[86] Gougelet A , Colnot S . MicroRNA‐feedback loop as a key modulator of liver tumorigenesis and inflammation. World J Gastroenterol. 2013;19(4):440‐444. 23382622 10.3748/wjg.v19.i4.440 PMC3558567

[87] Chen W , Daines MO , Khurana Hershey GK . Turning off signal transducer and activator of transcription (STAT): the negative regulation of STAT signaling. J Allergy Clin Immunol. 2004;114(3):476‐489. quiz 490. 15356544 10.1016/j.jaci.2004.06.042

[88] Gupta SC , Phromnoi K , Aggarwal BB . Morin inhibits STAT3 tyrosine 705 phosphorylation in tumor cells through activation of protein tyrosine phosphatase SHP1. Biochem Pharmacol. 2013;85(7):898‐912. 23279849 10.1016/j.bcp.2012.12.018

[89] Yang Y , Jiang B , Huo Y , et al. Shp2 suppresses PyMT‐induced transformation in mouse fibroblasts by inhibiting Stat3 activity. Virology. 2011;409(2):204‐210. 21056449 10.1016/j.virol.2010.09.032 PMC3008596

[90] Kumar V , Cheng P , Condamine T , et al. CD45 phosphatase inhibits STAT3 transcription factor activity in myeloid cells and promotes tumor‐associated macrophage differentiation. Immunity. 2016;44(2):303‐315. 26885857 10.1016/j.immuni.2016.01.014 PMC4759655

[91] Lee H , Kim M , Baek M , et al. Targeted disruption of TC‐PTP in the proliferative compartment augments STAT3 and AKT signaling and skin tumor development. Sci Rep. 2017;7:45077. 28322331 10.1038/srep45077 PMC5359614

[92] Lee JH , Mohan CD , Shanmugam MK , et al. Vitexin abrogates invasion and survival of hepatocellular carcinoma cells through targeting STAT3 signaling pathway. Biochimie. 2020;175:58‐68. 32445654 10.1016/j.biochi.2020.05.006

[93] Kim M , Morales LD , Jang I‐S , Cho Y‐Y , Kim DJ . Protein tyrosine phosphatases as potential regulators of STAT3 signaling. Int J Mol Sci. 2018;19(9):2708. 10.3390/ijms19092708 PMC6164089 30208623

[94] Lin Y , Zhou X , Yang K , et al. Protein tyrosine phosphatase receptor type D gene promotes radiosensitivity via STAT3 dephosphorylation in nasopharyngeal carcinoma. Oncogene. 2021;40(17):3101‐3117. 33824475 10.1038/s41388-021-01768-8 PMC8084736

[95] Durham GA , Williams JJ , Nasim MT , Palmer TM . Targeting SOCS proteins to control JAK‐STAT signalling in disease. Trends Pharmacol Sci. 2019;40(5):298‐308. 30948191 10.1016/j.tips.2019.03.001

[96] Yoshikawa H , Matsubara K , Qian GS , et al. SOCS‐1, a negative regulator of the JAK/STAT pathway, is silenced by methylation in human hepatocellular carcinoma and shows growth‐suppression activity. Nat Genet. 2001;28(1):29‐35. 11326271 10.1038/ng0501-29

[97] Neuwirt H , Puhr M , Santer FR , et al. Suppressor of cytokine signaling (SOCS)‐1 is expressed in human prostate cancer and exerts growth‐inhibitory function through down‐regulation of cyclins and cyclin‐dependent kinases. Am J Pathol. 2009;174(5):1921‐1930. 19342366 10.2353/ajpath.2009.080751 PMC2671279

[98] Wu M , Song D , Li H , et al. Negative regulators of STAT3 signaling pathway in cancers. Cancer Manag Res. 2019;11:4957‐4969. 31213912 10.2147/CMAR.S206175 PMC6549392

[99] Ma J , Yang Y , Fu Y , et al. PIAS3‐mediated feedback loops promote chronic colitis‐associated malignant transformation. Theranostics. 2018;8(11):3022‐3037. 29896300 10.7150/thno.23046 PMC5996365

[100] Lee JH , Kim C , Sethi G , Ahn KS . Brassinin inhibits STAT3 signaling pathway through modulation of PIAS‐3 and SOCS‐3 expression and sensitizes human lung cancer xenograft in nude mice to paclitaxel. Oncotarget. 2015;6(8):6386‐6405. 25788267 10.18632/oncotarget.3443 PMC4467444

[101] Saydmohammed M , Joseph D , Syed V . Curcumin suppresses constitutive activation of STAT‐3 by up‐regulating protein inhibitor of activated STAT‐3 (PIAS‐3) in ovarian and endometrial cancer cells. J Cell Biochem. 2010;110(2):447‐456. 20235152 10.1002/jcb.22558

[102] Jiang M , Zhang W , Zhang R , et al. Cancer exosome‐derived miR‐9 and miR‐181a promote the development of early‐stage MDSCs via interfering with SOCS3 and PIAS3 respectively in breast cancer. Oncogene. 2020;39(24):4681‐4694. 32398867 10.1038/s41388-020-1322-4

[103] Tan J , Xiang L , Xu G . LncRNA MEG3 suppresses migration and promotes apoptosis by sponging miR‐548d‐3p to modulate JAK‐STAT pathway in oral squamous cell carcinoma. IUBMB Life. 2019;71(7):882‐890. 30809930 10.1002/iub.2012

[104] Sharma U , Barwal TS , Khandelwal A , et al. LncRNA ZFAS1 inhibits triple‐negative breast cancer by targeting STAT3. Biochimie. 2021;182:99‐107. 33429003 10.1016/j.biochi.2020.12.026

[105] Wang Y , Wu S , Zhu X , et al. LncRNA‐encoded polypeptide ASRPS inhibits triple‐negative breast cancer angiogenesis. J Exp Med. 2020;217(3):jem.20190950. 10.1084/jem.20190950 PMC7062514 31816634

[106] Najm A , Masson FM , Preuss P , et al. MicroRNA‐17‐5p reduces inflammation and bone erosions in mice with collagen‐induced arthritis and directly targets the JAK/STAT pathway in rheumatoid arthritis fibroblast‐like synoviocytes. Arthritis Rheumatol. 2020;72(12):2030‐2039. 32683798 10.1002/art.41441

[107] Carraro G , El‐Hashash A , Guidolin D , et al. miR‐17 family of microRNAs controls FGF10‐mediated embryonic lung epithelial branching morphogenesis through MAPK14 and STAT3 regulation of E‐Cadherin distribution. Dev Biol. 2009;333(2):238‐250. 19559694 10.1016/j.ydbio.2009.06.020 PMC2735610

[108] Zhang H , Cai K , Wang J , et al. MiR‐7, inhibited indirectly by lincRNA HOTAIR, directly inhibits SETDB1 and reverses the EMT of breast cancer stem cells by downregulating the STAT3 pathway. Stem Cells. 2014;32(11):2858‐2868. 25070049 10.1002/stem.1795

[109] Xiao D , Cui X , Wang X . LncRNA PTCSC3 inhibits cell proliferation in laryngeal squamous cell carcinoma by down‐regulating lncRNA HOTAIR. Biosci Rep. 2019;39(6): BSR20182362. 10.1042/BSR20182362 PMC6597852 31171714

[110] Jin S , Yang X , Li J , Yang W , Ma H , Zhang Z . p53‐targeted lincRNA‐p21 acts as a tumor suppressor by inhibiting JAK2/STAT3 signaling pathways in head and neck squamous cell carcinoma. Mol Cancer. 2019;18(1):38. 30857539 10.1186/s12943-019-0993-3 PMC6410525

[111] Hanahan D , Weinberg RA . The hallmarks of cancer. Cell. 2000;100(1):57‐70. 10647931 10.1016/s0092-8674(00)81683-9

[112] Hanahan D , Weinberg RA . Hallmarks of cancer: the next generation. Cell. 2011;144(5):646‐674. 21376230 10.1016/j.cell.2011.02.013

[113] Hanahan D . Hallmarks of cancer: new dimensions. Cancer Discov. 2022;12(1):31‐46. 35022204 10.1158/2159-8290.CD-21-1059

[114] Pines J . Cyclins, CDKs and cancer. Semin Cancer Biol. 1995;6(2):63‐72. 7647308 10.1006/scbi.1995.0009

[115] Mittnacht S , Boshoff C . Viral cyclins. Rev Med Virol. 2000;10(3):175‐184. 10815028 10.1002/(sici)1099-1654(200005/06)10:3<175::aid-rmv283>3.0.co;2-f

[116] Deshpande A , Sicinski P , Hinds PW . Cyclins and cdks in development and cancer: a perspective. Oncogene. 2005;24(17):2909‐2915. 15838524 10.1038/sj.onc.1208618

[117] Deepak Roshan VG , Sinto MS , Thomas S , Kannan S . Cyclin D1 overexpression associated with activation of STAT3 in oral carcinoma patients from South India. J Cancer Res Ther. 2018;14(2):403‐408. 29516928 10.4103/0973-1482.188295

[118] Luo J , Yan R , He X , He J . Constitutive activation of STAT3 and cyclin D1 overexpression contribute to proliferation, migration and invasion in gastric cancer cells. Am J Transl Res. 2017;9(12):5671. 29312519 PMC5752917

[119] Yang JG , Lu R , Ye XJ , Zhang J , Tan YQ , Zhou G . Icaritin reduces oral squamous cell carcinoma progression via the inhibition of STAT3 signaling. Int J Mol Sci. 2017;18(1):132. 10.3390/ijms18010132 PMC5297765 28085115

[120] Adams JM , Cory S . The Bcl‐2 apoptotic switch in cancer development and therapy. Oncogene. 2007;26(9):1324‐1337. 17322918 10.1038/sj.onc.1210220 PMC2930981

[121] Warren CFA , Wong‐Brown MW , Bowden NA . BCL‐2 family isoforms in apoptosis and cancer. Cell Death Dis. 2019;10(3):177. 30792387 10.1038/s41419-019-1407-6 PMC6384907

[122] Bessou M , Lopez J , Gadet R , et al. The apoptosis inhibitor Bcl‐xL controls breast cancer cell migration through mitochondria‐dependent reactive oxygen species production. Oncogene. 2020;39(15):3056‐3074. 32066881 10.1038/s41388-020-1212-9

[123] Lee EF , Harris TJ , Tran S , et al. BCL‐XL and MCL‐1 are the key BCL‐2 family proteins in melanoma cell survival. Cell Death Dis. 2019;10(5):1‐14. 10.1038/s41419-019-1568-3 PMC6482196 31019203

[124] Jiao Y , Ding H , Huang S , et al. Bcl‐XL and Mcl‐1 upregulation by calreticulin promotes apoptosis resistance of fibroblast‐like synoviocytes via activation of PI3K/Akt and STAT3 pathways in rheumatoid arthritis. Clin Exp Rheumatol. 2018;36(5):841‐849. 29652658

[125] Catlett‐Falcone R , Landowski TH , Oshiro MM , et al. Constitutive activation of Stat3 signaling confers resistance to apoptosis in human U266 myeloma cells. Immunity. 1999;10(1):105‐115. 10023775 10.1016/s1074-7613(00)80011-4

[126] Oh HN , Oh KB , Lee MH , et al. JAK2 regulation by licochalcone H inhibits the cell growth and induces apoptosis in oral squamous cell carcinoma. Phytomedicine. 2019;52:60‐69. 30599913 10.1016/j.phymed.2018.09.180

[127] Yasuda T , Koiwa M , Yonemura A , et al. Inflammation‐driven senescence‐associated secretory phenotype in cancer‐associated fibroblasts enhances peritoneal dissemination. Cell Rep. 2021;34(8):108779. 33626356 10.1016/j.celrep.2021.108779

[128] Zhao H , Wu L , Yan G , et al. Inflammation and tumor progression: signaling pathways and targeted intervention. Signal Transduc Target Ther. 2021;6(1):263. 10.1038/s41392-021-00658-5 PMC8273155 34248142

[129] Powell DR , Huttenlocher A . Neutrophils in the tumor microenvironment. Trends Immunol. 2016;37(1):41‐52. 26700397 10.1016/j.it.2015.11.008 PMC4707100

[130] Raposo TP , Beirão BC , Pang LY , Queiroga FL , Argyle DJ . Inflammation and cancer: till death tears them apart. Vet J. 2015;205(2):161‐174. 25981934 10.1016/j.tvjl.2015.04.015

[131] Huang L , Guo Y , Liu S , et al. Targeting regulatory T cells for immunotherapy in melanoma. Mol Biomed. 2021;2(1):11. 34806028 10.1186/s43556-021-00038-z PMC8591697

[132] Sun C , Mezzadra R , Schumacher TN . Regulation and function of the PD‐L1 checkpoint. Immunity. 2018;48(3):434‐452. 29562194 10.1016/j.immuni.2018.03.014 PMC7116507

[133] Bu L , Yu G , Wu L , et al. STAT3 induces immunosuppression by upregulating PD‐1/PD‐L1 in HNSCC. J Dent Res. 2017;96(9):1027‐1034. 28605599 10.1177/0022034517712435 PMC6728673

[134] Xu L , Chen X , Shen M , et al. Inhibition of IL‐6‐JAK/Stat3 signaling in castration‐resistant prostate cancer cells enhances the NK cell‐mediated cytotoxicity via alteration of PD‐L1/NKG2D ligand levels. Mol Oncol. 2018;12(3):269‐286. 28865178 10.1002/1878-0261.12135 PMC5830627

[135] Cheng Y , Li H , Deng Y , et al. Cancer‐associated fibroblasts induce PDL1+ neutrophils through the IL6‐STAT3 pathway that foster immune suppression in hepatocellular carcinoma. Cell Death Dis. 2018;9(4):1‐11. 29556041 10.1038/s41419-018-0458-4 PMC5859264

[136] Sun R , Xiong Y , Liu H , et al. Tumor‐associated neutrophils suppress antitumor immunity of NK cells through the PD‐L1/PD‐1 axis. Transl Oncol. 2020;13(10):100825. 32698059 10.1016/j.tranon.2020.100825 PMC7372151

[137] Italiani P , Boraschi D . From monocytes to M1/M2 macrophages: phenotypical vs. functional differentiation. Front Immunol. 2014;5:514. 25368618 10.3389/fimmu.2014.00514 PMC4201108

[138] Fu X‐L , Duan W , Su C‐Y , et al. Interleukin 6 induces M2 macrophage differentiation by STAT3 activation that correlates with gastric cancer progression. Cancer Immunol Immunother. 2017;66(12):1597‐1608. 28828629 10.1007/s00262-017-2052-5 PMC11028627

[139] Yin Z , Ma T , Lin Y , et al. IL‐6/STAT3 pathway intermediates M1/M2 macrophage polarization during the development of hepatocellular carcinoma. J Cell Biochem. 2018;119(11):9419‐9432. 30015355 10.1002/jcb.27259

[140] Di Conza G , Tsai CH , Gallart‐Ayala H , et al. Tumor‐induced reshuffling of lipid composition on the endoplasmic reticulum membrane sustains macrophage survival and pro‐tumorigenic activity. Nat Immunol. 2021;22(11):1403‐1415. 34686867 10.1038/s41590-021-01047-4 PMC7611917

[141] Zhang X , Zeng Y , Qu Q , et al. PD‐L1 induced by IFN‐γ from tumor‐associated macrophages via the JAK/STAT3 and PI3K/AKT signaling pathways promoted progression of lung cancer. Int J Clin Oncol. 2017;22(6):1026‐1033. 28748356 10.1007/s10147-017-1161-7

[142] Vasquez‐Dunddel D , Pan F , Zeng Q , et al. STAT3 regulates arginase‐I in myeloid‐derived suppressor cells from cancer patients. J Clin Invest. 2013;123(4):1580‐1589. 23454751 10.1172/JCI60083 PMC3613901

[143] Wu AA , Drake V , Huang HS , Chiu S , Zheng L . Reprogramming the tumor microenvironment: tumor‐induced immunosuppressive factors paralyze T cells. Oncoimmunology. 2015;4(7):e1016700. 26140242 10.1080/2162402X.2015.1016700 PMC4485788

[144] Li K , Shi H , Zhang B , et al. Myeloid‐derived suppressor cells as immunosuppressive regulators and therapeutic targets in cancer. Signal Transduc Target Ther. 2021;6(1):362. 10.1038/s41392-021-00670-9 PMC8497485 34620838

[145] Waldner MJ , Neurath MF . Master regulator of intestinal disease: IL‐6 in chronic inflammation and cancer development. Semin Immunol. 2014;26(1):75‐79. 24447345 10.1016/j.smim.2013.12.003

[146] Kortylewski M , Kujawski M , Wang T , et al. Inhibiting Stat3 signaling in the hematopoietic system elicits multicomponent antitumor immunity. Nat Med. 2005;11(12):1314‐1321. 16288283 10.1038/nm1325

[147] Jewett A , Head C , Cacalano NA . Emerging mechanisms of immunosuppression in oral cancers. J Dent Res. 2006;85(12):1061‐1073. 17122156 10.1177/154405910608501201

[148] Priceman Saul J , Shen S , Wang L , et al. S1PR1 is crucial for accumulation of regulatory T cells in tumors via STAT3. Cell Rep. 2014;6(6):992‐999. 24630990 10.1016/j.celrep.2014.02.016 PMC3988983

[149] Hanna BS , Llaó‐Cid L , Iskar M , et al. Interleukin‐10 receptor signaling promotes the maintenance of a PD‐1int TCF‐1+ CD8+ T cell population that sustains anti‐tumor immunity. Immunity. 2021;54(12):2825‐2841.e10. 34879221 10.1016/j.immuni.2021.11.004

[150] Gupta MK , Qin RY . Mechanism and its regulation of tumor‐induced angiogenesis. World J Gastroenterol. 2003;9(6):1144‐1155. 12800214 10.3748/wjg.v9.i6.1144 PMC4611774

[151] Mathonnet M , Perraud A , Christou N , et al. Hallmarks in colorectal cancer: angiogenesis and cancer stem‐like cells. World J Gastroenterol. 2014;20(15):4189‐4196. 24764657 10.3748/wjg.v20.i15.4189 PMC3989955

[152] Weis SM , Cheresh DA . Tumor angiogenesis: molecular pathways and therapeutic targets. Nat Med. 2011;17(11):1359‐1370. 22064426 10.1038/nm.2537

[153] Niu G , Wright KL , Huang M , et al. Constitutive Stat3 activity up‐regulates VEGF expression and tumor angiogenesis. Oncogene. 2002;21(13):2000‐2008. 11960372 10.1038/sj.onc.1205260

[154] Tamura R , Tanaka T , Akasaki Y , Murayama Y , Yoshida K , Sasaki H . The role of vascular endothelial growth factor in the hypoxic and immunosuppressive tumor microenvironment: perspectives for therapeutic implications. Med Oncol. 2019;37(1):2. 31713115 10.1007/s12032-019-1329-2

[155] Tong F , Xiong CJ , Wei CH , et al. Hypo‐fractionation radiotherapy normalizes tumor vasculature in non‐small cell lung cancer xenografts through the p‐STAT3/HIF‐1 alpha signaling pathway. Ther Adv Med Oncol. 2020;12:1758835920965853. 33193827 10.1177/1758835920965853 PMC7605032

[156] Zhang P‐C , Liu X , Li M‐M , et al. AT‐533, a novel Hsp90 inhibitor, inhibits breast cancer growth and HIF‐1α/VEGF/VEGFR‐2‐mediated angiogenesis in vitro and in vivo. Biochem Pharmacol. 2020;172:113771. 31863779 10.1016/j.bcp.2019.113771

[157] Liu P , Atkinson SJ , Akbareian SE , et al. Sulforaphane exerts anti‐angiogenesis effects against hepatocellular carcinoma through inhibition of STAT3/HIF‐1α/VEGF signalling. Sci Rep. 2017;7(1):12651. 28978924 10.1038/s41598-017-12855-w PMC5627255

[158] Zhang N , Zhang M , Wang Z , Gao W , Sun Z‐G . Activated STAT3 could reduce survival in patients with esophageal squamous cell carcinoma by up‐regulating VEGF and cyclin D1 expression. J Cancer. 2020;11(7):1859‐1868. 32194797 10.7150/jca.38798 PMC7052867

[159] Quintero‐Fabián S , Arreola R , Becerril‐Villanueva E , et al. Role of matrix metalloproteinases in angiogenesis and cancer. Front Oncol. 2019;9:1370. 31921634 10.3389/fonc.2019.01370 PMC6915110

[160] Jodele S , Chantrain CF , Blavier L , et al. The contribution of bone marrow‐derived cells to the tumor vasculature in neuroblastoma is matrix metalloproteinase‐9 dependent. Cancer Res. 2005;65(8):3200‐3208. 15833851 10.1158/0008-5472.CAN-04-3770

[161] Roy R , Dagher A , Butterfield C , Moses MA . ADAM12 Is a novel regulator of tumor angiogenesis via STAT3 signaling. Mol Cancer Res. 2017;15(11):1608‐1622. 28765266 10.1158/1541-7786.MCR-17-0188 PMC5668165

[162] Kujawski M , Kortylewski M , Lee H , Herrmann A , Kay H , Yu H . Stat3 mediates myeloid cell‐dependent tumor angiogenesis in mice. J Clin Invest. 2008;118(10):3367‐3377. 18776941 10.1172/JCI35213 PMC2528912

[163] Xu Q , Ma H , Chang H , Feng Z , Zhang C , Yang X . The interaction of interleukin‐8 and PTEN inactivation promotes the malignant progression of head and neck squamous cell carcinoma via the STAT3 pathway. Cell Death Dis. 2020;11(5):405. 32471980 10.1038/s41419-020-2627-5 PMC7260373

[164] Ko HS , Park BJ , Choi SK , et al. STAT3 and ERK signaling pathways are implicated in the invasion activity by oncostatin M through induction of matrix metalloproteinases 2 and 9. Yonsei Med J. 2016;57(3):761‐768. 26996579 10.3349/ymj.2016.57.3.761 PMC4800369

[165] Park SY , Jang WJ , Yi EY , et al. Melatonin suppresses tumor angiogenesis by inhibiting HIF‐1alpha stabilization under hypoxia. J Pineal Res. 2010;48(2):178‐184. 20449875 10.1111/j.1600-079x.2009.00742.x

[166] Wu F , Yang J , Liu J , et al. Signaling pathways in cancer‐associated fibroblasts and targeted therapy for cancer. Signal Transduc Target Ther. 2021;6(1):218. 10.1038/s41392-021-00641-0 PMC8190181 34108441

[167] Smith A , Teknos TN , Pan Q . Epithelial to mesenchymal transition in head and neck squamous cell carcinoma. Oral Oncol. 2013;49(4):287‐292. 23182398 10.1016/j.oraloncology.2012.10.009 PMC3586749

[168] Pastushenko I , Blanpain C . EMT transition states during tumor progression and metastasis. Trends Cell Biol. 2019;29(3):212‐226. 30594349 10.1016/j.tcb.2018.12.001

[169] Chao J , Zhao S , Sun H . Dedifferentiation of hepatocellular carcinoma: molecular mechanisms and therapeutic implications. Am J Transl Res. 2020;12(5):2099‐2109. 32509204 PMC7269980

[170] Taylor KM , Hiscox S , Nicholson RI . Zinc transporter LIV‐1: a link between cellular development and cancer progression. Trends Endocrinol Metab. 2004;15(10):461‐463. 15541644 10.1016/j.tem.2004.10.003

[171] Zhang W , Zhang J , Zhang Z , et al. Overexpression of indoleamine 2,3‐dioxygenase 1 promotes epithelial‐mesenchymal transition by activation of the IL‐6/STAT3/PD‐L1 pathway in bladder cancer. Transl Oncol. 2019;12(3):485‐492. 30594037 10.1016/j.tranon.2018.11.012 PMC6307990

[172] Sun SS , Zhou X , Huang YY , et al. Targeting STAT3/miR‐21 axis inhibits epithelial‐mesenchymal transition via regulating CDK5 in head and neck squamous cell carcinoma. Mol Cancer. 2015;14:213. 26690371 10.1186/s12943-015-0487-x PMC4687320

[173] Zhao M , Hu X , Xu Y , et al. Targeting of EZH2 inhibits epithelial‑mesenchymal transition in head and neck squamous cell carcinoma via regulating the STAT3/VEGFR2 axis. Int J Oncol. 2019;55(5):1165‐1175. 31545422 10.3892/ijo.2019.4880

[174] Yadav A , Kumar B , Datta J , Teknos TN , Kumar P . IL‐6 promotes head and neck tumor metastasis by inducing epithelial‐mesenchymal transition via the JAK‐STAT3‐SNAIL signaling pathway. Mol Cancer Res. 2011;9(12):1658‐1667. 21976712 10.1158/1541-7786.MCR-11-0271 PMC3243808

[175] Ligorio M , Sil S , Malagon‐Lopez J , et al. Stromal microenvironment shapes the intratumoral architecture of pancreatic cancer. Cell. 2019;178(1):160‐175.e27. 31155233 10.1016/j.cell.2019.05.012 PMC6697165

[176] Chen YW , Chen KH , Huang PI , et al. Cucurbitacin I suppressed stem‐like property and enhanced radiation‐induced apoptosis in head and neck squamous carcinoma–derived CD44(+)ALDH1(+) cells. Mol Cancer Ther. 2010;9(11):2879‐2892. 21062915 10.1158/1535-7163.MCT-10-0504

[177] Wu TF , Chen L , Bu LL , Gao J , Zhang WF , Jia J . CD44+ cancer cell‐induced metastasis: a feasible neck metastasis model. Eur J Pharm Sci. 2017;101:243‐250. 28215944 10.1016/j.ejps.2017.02.020

[178] Reya T , Morrison SJ , Clarke MF , Weissman IL . Stem cells, cancer, and cancer stem cells. Nature. 2001;414(6859):105‐111. 11689955 10.1038/35102167

[179] Huang WC , Jang TH , Tung SL , Yen TC , Chan SH , Wang LH . A novel miR‐365‐3p/EHF/keratin 16 axis promotes oral squamous cell carcinoma metastasis, cancer stemness and drug resistance via enhancing beta5‐integrin/c‐met signaling pathway. J Exp Clin Cancer Res. 2019;38(1):89. 30782177 10.1186/s13046-019-1091-5 PMC6381632

[180] Kim S‐Y , Kang JW , Song X , et al. Role of the IL‐6‐JAK1‐STAT3‐Oct‐4 pathway in the conversion of non‐stem cancer cells into cancer stem‐like cells. Cell Signalling. 2013;25(4):961‐969. 23333246 10.1016/j.cellsig.2013.01.007 PMC3595341

[181] Yang J , Liao D , Chen C , et al. Tumor‐associated macrophages regulate murine breast cancer stem cells through a novel paracrine EGFR/Stat3/Sox‐2 signaling pathway. Stem Cells. 2013;31(2):248‐258. 23169551 10.1002/stem.1281

[182] Ning Y , Cui Y , Li X , et al. Co‐culture of ovarian cancer stem‐like cells with macrophages induced SKOV3 cells stemness via IL‐8/STAT3 signaling. Biomed Pharmacother. 2018;103:262‐271. 29656182 10.1016/j.biopha.2018.04.022

[183] Tanabe S , Quader S , Cabral H , Ono R . Interplay of EMT and CSC in cancer and the potential therapeutic strategies. Front Pharmacol. 2020;11:904. 10.3389/fphar.2020.00904 PMC7311659 32625096

[184] Dave B , Mittal V , Tan NM , Chang JC . Epithelial‐mesenchymal transition, cancer stem cells and treatment resistance. Breast Cancer Res. 2012;14(1):1‐5. 10.1186/bcr2938 PMC3496111 22264257

[185] Zhou P , Li B , Liu F , et al. The epithelial to mesenchymal transition (EMT) and cancer stem cells: implication for treatment resistance in pancreatic cancer. Mol Cancer. 2017;16(1):1‐11. 28245823 10.1186/s12943-017-0624-9 PMC5331747

[186] Smigiel JM , Parameswaran N , Jackson MW . Potent EMT and CSC phenotypes are induced by oncostatin‐M in pancreatic cancer. Mol Cancer Res. 2017;15(4):478‐488. 28053127 10.1158/1541-7786.MCR-16-0337 PMC5380554

[187] Jin W . Role of JAK/STAT3 signaling in the regulation of metastasis, the transition of cancer stem cells, and chemoresistance of cancer by epithelial–mesenchymal transition. Cells. 2020;9(1):217. 10.3390/cells9010217 PMC7017057 31952344

[188] Junk DJ , Bryson BL , Smigiel JM , Parameswaran N , Bartel CA , Jackson MW . Oncostatin M promotes cancer cell plasticity through cooperative STAT3‐SMAD3 signaling. Oncogene. 2017;36(28):4001‐4013. 28288136 10.1038/onc.2017.33 PMC5509502

[189] Zhao C , Li H , Lin HJ , Yang S , Lin J , Liang G . Feedback activation of STAT3 as a cancer drug‐resistance mechanism. Trends Pharmacol Sci. 2016;37(1):47‐61. 26576830 10.1016/j.tips.2015.10.001

[190] Liu WH , Chen MT , Wang ML , et al. Cisplatin‐selected resistance is associated with increased motility and stem‐like properties via activation of STAT3/Snail axis in atypical teratoid/rhabdoid tumor cells. Oncotarget. 2015;6(3):1750‐1768. 25638155 10.18632/oncotarget.2737 PMC4359329

[191] Zhang JL , Liu XZ , Wang PY , et al. Targeting HCCR expression resensitizes gastric cancer cells to chemotherapy via down‐regulating the activation of STAT3. Sci Rep. 2016;6:24196. 27052330 10.1038/srep24196 PMC4823702

[192] Huang W , Zhong Z , Luo C , et al. The miR‐26a/AP‐2α/Nanog signaling axis mediates stem cell self‐renewal and temozolomide resistance in glioma. Theranostics. 2019;9(19):5497‐5516. 31534499 10.7150/thno.33800 PMC6735392

[193] Su JC , Tseng PH , Wu SH , et al. SC‐2001 overcomes STAT3‐mediated sorafenib resistance through RFX‐1/SHP‐1 activation in hepatocellular carcinoma. Neoplasia. 2014;16(7):595‐605. 25047655 10.1016/j.neo.2014.06.005 PMC4198826

[194] Liberti MV , Locasale JW . The Warburg effect: how does it benefit cancer cells? Trends Biochem Sci. 2016;41(3):211‐218. 26778478 10.1016/j.tibs.2015.12.001 PMC4783224

[195] DeBerardinis RJ , Chandel NS . We need to talk about the Warburg effect. Nat Metab. 2020;2(2):127‐129. 32694689 10.1038/s42255-020-0172-2

[196] Zhu S , Dong Z , Ke X et‐al. The roles of sirtuins family in cell metabolism during tumor development. Semin Cancer Biol.. 2019;57:59‐71. 30453040 10.1016/j.semcancer.2018.11.003

[197] Bi YH , Han WQ , Li RF , et al. Signal transducer and activator of transcription 3 promotes the Warburg effect possibly by inducing pyruvate kinase M2 phosphorylation in liver precancerous lesions. World J Gastroenterol. 2019;25(16):1936‐1949. 31086462 10.3748/wjg.v25.i16.1936 PMC6487376

[198] Li Q , Zhang D , Chen X , et al. Nuclear PKM2 contributes to gefitinib resistance via upregulation of STAT3 activation in colorectal cancer. Sci Rep. 2015;5:16082. 26542452 10.1038/srep16082 PMC4635355

[199] Guan M , Tong Y , Guan M , et al. Lapatinib inhibits breast cancer cell proliferation by influencing PKM2 expression. Technol Cancer Res Treat. 2018;17:1533034617749418. 29343208 10.1177/1533034617749418 PMC5784572

[200] Demaria M , Poli V . PKM2, STAT3 and HIF‐1α: the Warburg's vicious circle. Jak‐stat. 2012;1(3):194‐196. 24058770 10.4161/jkst.20662 PMC3670244

[201] Fan M , Sun W , Gu X , et al. The critical role of STAT3 in biogenesis of tumor‐derived exosomes with potency of inducing cancer cachexia in vitro and in vivo. Oncogene. 2022;41:1050‐1062. 35034093 10.1038/s41388-021-02151-3

[202] Pu Z , Xu M , Yuan X , Xie H , Zhao J . Circular RNA circCUL3 accelerates the Warburg effect progression of gastric cancer through regulating the STAT3/HK2 axis. Mol Ther Nucleic Acids. 2020;22:310‐318. 33230436 10.1016/j.omtn.2020.08.023 PMC7527579

[203] Yu GT , Mao L , Wu L , et al. Inhibition of SRC family kinases facilitates anti‐CTLA4 immunotherapy in head and neck squamous cell carcinoma. Cell Mol Life Sci. 2018;75(22):4223‐4234. 29955905 10.1007/s00018-018-2863-3 PMC11105240

[204] Bauman JE , Duvvuri U , Gooding WE , et al. Randomized, placebo‐controlled window trial of EGFR, Src, or combined blockade in head and neck cancer. JCI Insight. 2017;2(6):e90449. 28352657 10.1172/jci.insight.90449 PMC5358497

[205] Kim DW , Saussele S , Williams LA , et al. Outcomes of switching to dasatinib after imatinib‐related low‐grade adverse events in patients with chronic myeloid leukemia in chronic phase: the DASPERSE study. Ann Hematol. 2018;97(8):1357‐1367. 29556695 10.1007/s00277-018-3295-8 PMC6018625

[206] Finn RS , Bengala C , Ibrahim N , et al. Dasatinib as a single agent in triple‐negative breast cancer: results of an open‐label phase 2 study. Clin Cancer Res. 2011;17(21):6905‐6913. 22028489 10.1158/1078-0432.CCR-11-0288

[207] Foà R , Bassan R , Vitale A , et al. Dasatinib‐Blinatumomab for Ph‐positive acute lymphoblastic leukemia in adults. N Engl J Med. 2020;383(17):1613‐1623. 33085860 10.1056/NEJMoa2016272

[208] Yu EY , Wilding G , Posadas E , et al. Phase II study of dasatinib in patients with metastatic castration‐resistant prostate cancer. Clin Cancer Res. 2009;15(23):7421‐7428. 19920114 10.1158/1078-0432.CCR-09-1691 PMC3394097

[209] Bonner JA , Yang ES , Trummell HQ , Nowsheen S , Willey CD , Raisch KP . Inhibition of STAT‐3 results in greater cetuximab sensitivity in head and neck squamous cell carcinoma. Radiother Oncol. 2011;99(3):339‐343. 21704410 10.1016/j.radonc.2011.05.070

[210] Park SH , Jo MJ , Kim BR , et al. Sonic hedgehog pathway activation is associated with cetuximab resistance and EPHB3 receptor induction in colorectal cancer. Theranostics. 2019;9(8):2235‐2251. 31149041 10.7150/thno.30678 PMC6531304

[211] Kopetz S , Grothey A , Yaeger R , et al. Encorafenib, binimetinib, and cetuximab in BRAF V600E‐mutated colorectal cancer. N Engl J Med. 2019;381(17):1632‐1643. 31566309 10.1056/NEJMoa1908075

[212] Kopetz S , Guthrie KA , Morris VK , et al. Randomized trial of irinotecan and cetuximab with or without vemurafenib in BRAF‐mutant metastatic colorectal cancer (SWOG S1406). J Clin Oncol. 2021;39(4):285‐294. 33356422 10.1200/JCO.20.01994 PMC8462593

[213] Burtness B , Harrington KJ , Greil R , et al. Pembrolizumab alone or with chemotherapy versus cetuximab with chemotherapy for recurrent or metastatic squamous cell carcinoma of the head and neck (KEYNOTE‐048): a randomised, open‐label, phase 3 study. Lancet. 2019;394(10212):1915‐1928. 31679945 10.1016/S0140-6736(19)32591-7

[214] Sen M , Pollock NI , Black J , et al. JAK kinase inhibition abrogates STAT3 activation and head and neck squamous cell carcinoma tumor growth. Neoplasia. 2015;17(3):256‐264. 25810010 10.1016/j.neo.2015.01.003 PMC4372647

[215] Wang SW , Hu J , Guo QH , et al. AZD1480, a JAK inhibitor, inhibits cell growth and survival of colorectal cancer via modulating the JAK2/STAT3 signaling pathway. Oncol Rep. 2014;32(5):1991‐1998. 25216185 10.3892/or.2014.3477

[216] Stover DG , Gil Del Alcazar CR , Brock J , et al. Phase II study of ruxolitinib, a selective JAK1/2 inhibitor, in patients with metastatic triple‐negative breast cancer. NPJ Breast Cancer. 2018;4:10. 29761158 10.1038/s41523-018-0060-z PMC5935675

[217] Park JS , Hong MH , Chun YJ , Kim HR , Cho BC . A phase Ib study of the combination of afatinib and ruxolitinib in EGFR mutant NSCLC with progression on EGFR‐TKIs. Lung Cancer. 2019;134:46‐51. 31319994 10.1016/j.lungcan.2019.05.030

[218] Hurwitz HI , Uppal N , Wagner SA , et al. Randomized, double‐blind, phase II study of ruxolitinib or placebo in combination with capecitabine in patients with metastatic pancreatic cancer for whom therapy with gemcitabine has failed. J Clin Oncol. 2015;33(34):4039‐4047. 26351344 10.1200/JCO.2015.61.4578 PMC5089161

[219] Bilori B , Thota S , Clemente MJ , et al. Tofacitinib as a novel salvage therapy for refractory T‐cell large granular lymphocytic leukemia. Leukemia. 2015;29(12):2427‐2429. 26449659 10.1038/leu.2015.280

[220] Pouyfung P , Choonate S , Wongnoppavich A , Rongnoparut P , Chairatvit K . Anti‐proliferative effect of 8α‐tigloyloxyhirsutinolide‐13‐O‐acetate (8αTGH) isolated from Vernonia cinerea on oral squamous cell carcinoma through inhibition of STAT3 and STAT2 phosphorylation. Phytomedicine. 2019;52:238‐246. 30599904 10.1016/j.phymed.2018.09.211

[221] Kumar B , Yadav A , Hideg K , Kuppusamy P , Teknos TN , Kumar P . A novel curcumin analog (H‐4073) enhances the therapeutic efficacy of cisplatin treatment in head and neck cancer. PLoS One. 2014;9(3):e93208. 24675768 10.1371/journal.pone.0093208 PMC3968069

[222] Mehta HJ , Patel V , Sadikot RT . Curcumin and lung cancer–a review. Target Oncol. 2014;9(4):295‐310. 24840628 10.1007/s11523-014-0321-1

[223] Zhou X , Ren Y , Liu A , et al. WP1066 sensitizes oral squamous cell carcinoma cells to cisplatin by targeting STAT3/miR‐21 axis. Sci Rep. 2014;4:7461. 25514838 10.1038/srep07461 PMC4268632

[224] Judd LM , Menheniott TR , Ling H , et al. Inhibition of the JAK2/STAT3 pathway reduces gastric cancer growth in vitro and in vivo. PLoS One. 2014;9(5):e95993. 24804649 10.1371/journal.pone.0095993 PMC4013079

[225] Sau S , Mondal SK , Kashaw SK , Iyer AK , Banerjee R . Combination of cationic dexamethasone derivative and STAT3 inhibitor (WP1066) for aggressive melanoma: a strategy for repurposing a phase I clinical trial drug. Mol Cell Biochem. 2017;436(1‐2):119‐136. 28585089 10.1007/s11010-017-3084-z

[226] Matsuoka Y , Nakayama H , Yoshida R , et al. IL‐6 controls resistance to radiation by suppressing oxidative stress via the Nrf2‐antioxidant pathway in oral squamous cell carcinoma. Br J Cancer. 2016;115(10):1234‐1244. 27736845 10.1038/bjc.2016.327 PMC5104896

[227] Alraouji NN , Al‐Mohanna FH , Ghebeh H , et al. Tocilizumab potentiates cisplatin cytotoxicity and targets cancer stem cells in triple‐negative breast cancer. Mol Carcinog. 2020;59(9):1041‐1051. 32537818 10.1002/mc.23234

[228] Liu FT , Jia L , Wang P , et al. CD126 and targeted therapy with tocilizumab in chronic lymphocytic leukemia. Clin Cancer Res. 2016;22(10):2462‐2469. 26712690 10.1158/1078-0432.CCR-15-1139

[229] Karkera J , Steiner H , Li W , et al. The anti‐interleukin‐6 antibody siltuximab down‐regulates genes implicated in tumorigenesis in prostate cancer patients from a phase I study. Prostate. 2011;71(13):1455‐1465. 21321981 10.1002/pros.21362

[230] Song L , Rawal B , Nemeth JA , Haura EB . JAK1 activates STAT3 activity in non‐small‐cell lung cancer cells and IL‐6 neutralizing antibodies can suppress JAK1‐STAT3 signaling. Mol Cancer Ther. 2011;10(3):481‐494. 21216930 10.1158/1535-7163.MCT-10-0502 PMC4084653

[231] Wu X , Cao Y , Xiao H , Li C , Lin J . Bazedoxifene as a novel GP130 inhibitor for pancreatic cancer therapy. Mol Cancer Ther. 2016;15(11):2609‐2619. 27535971 10.1158/1535-7163.MCT-15-0921 PMC5310670

[232] Curry J , Johnson J , Tassone P , et al. Metformin effects on head and neck squamous carcinoma microenvironment: window of opportunity trial. Laryngoscope. 2017;127(8):1808‐1815. 28185288 10.1002/lary.26489 PMC5515672

[233] Brown JR , Chan DK , Shank JJ , et al. Phase II clinical trial of metformin as a cancer stem cell‐targeting agent in ovarian cancer. JCI Insight. 2020;5(11):e133247. 10.1172/jci.insight.133247 PMC7308054 32369446

[234] Siddiquee KA , Gunning PT , Glenn M , et al. An oxazole‐based small‐molecule Stat3 inhibitor modulates Stat3 stability and processing and induces antitumor cell effects. ACS Chem Biol. 2007;2(12):787‐798. 18154266 10.1021/cb7001973

[235] Bu LL , Deng WW , Huang CF , Liu B , Zhang WF , Sun ZJ . Inhibition of STAT3 reduces proliferation and invasion in salivary gland adenoid cystic carcinoma. Am J Cancer Res. 2015;5(5):1751‐1761. 26175943 PMC4497441

[236] Bu LL , Yu GT , Deng WW , et al. Targeting STAT3 signaling reduces immunosuppressive myeloid cells in head and neck squamous cell carcinoma. Oncoimmunology. 2016;5(5):e1130206. 27467947 10.1080/2162402X.2015.1130206 PMC4910719

[237] Bu LL , Li YC , Yu GT , et al. Targeting phosphorylation of STAT3 delays tumor growth in HPV‐negative anal squamous cell carcinoma mouse model. Sci Rep. 2017;7(1):6629. 28747781 10.1038/s41598-017-06643-9 PMC5529522

[238] Song H , Wang R , Wang S , Lin J . A low‐molecular‐weight compound discovered through virtual database screening inhibits Stat3 function in breast cancer cells. Proc Natl Acad Sci U S A. 2005;102(13):4700‐4705. 15781862 10.1073/pnas.0409894102 PMC555708

[239] Pan Y , Zhou F , Zhang R , Claret FX . Correction: stat3 inhibitor stattic exhibits potent antitumor activity and induces chemo‐ and radio‐sensitivity in nasopharyngeal carcinoma. PLoS One. 2020;15(8):e0237943. 32790751 10.1371/journal.pone.0237943 PMC7425959

[240] Genini D , Brambilla L , Laurini E , et al. Mitochondrial dysfunction induced by a SH2 domain‐targeting STAT3 inhibitor leads to metabolic synthetic lethality in cancer cells. Proc Natl Acad Sci U S A. 2017;114(25):E4924‐E4933. 28584133 10.1073/pnas.1615730114 PMC5488915

[241] Ogura M , Uchida T , Terui Y , et al. Phase I study of OPB‐51602, an oral inhibitor of signal transducer and activator of transcription 3, in patients with relapsed/refractory hematological malignancies. Cancer Sci. 2015;106(7):896‐901. 25912076 10.1111/cas.12683 PMC4520642

[242] Wong AL , Soo RA , Tan DS , et al. Phase I and biomarker study of OPB‐51602, a novel signal transducer and activator of transcription (STAT) 3 inhibitor, in patients with refractory solid malignancies. Ann Oncol. 2015;26(5):998‐1005. 25609248 10.1093/annonc/mdv026

[243] Yoo C , Kang J , Lim HY , et al. Phase I dose‐finding study of OPB‐111077, a novel STAT3 inhibitor, in patients with advanced hepatocellular carcinoma. Cancer Res Treat. 2019;51(2):510‐518. 29898591 10.4143/crt.2018.226 PMC6473286

[244] Jung KH , Yoo W , Stevenson HL , et al. Multifunctional effects of a small‐molecule STAT3 inhibitor on NASH and hepatocellular carcinoma in mice. Clin Cancer Res. 2017;23(18):5537‐5546. 28533225 10.1158/1078-0432.CCR-16-2253 PMC5873583

[245] Di JX , Zhang HY . C188‐9, a small‐molecule STAT3 inhibitor, exerts an antitumor effect on head and neck squamous cell carcinoma. Anticancer Drugs. 2019;30(8):846‐853. 30870229 10.1097/CAD.0000000000000783

[246] Chen J , Bai L , Bernard D , et al. Structure‐based design of conformationally constrained, cell‐permeable STAT3 inhibitors. ACS Med Chem Lett. 2010;1(2):85‐89. 20596242 10.1021/ml100010j PMC2892918

[247] Leong PL , Andrews GA , Johnson DE , et al. Targeted inhibition of Stat3 with a decoy oligonucleotide abrogates head and neck cancer cell growth. Proc Natl Acad Sci U S A. 2003;100(7):4138‐4143. 12640143 10.1073/pnas.0534764100 PMC153061

[248] Carmicheal J , Kaur S , Batra SK , Ganti AK . Hunting for transcription factors: STAT3 decoy in non‐small cell lung cancer. Transl Lung Cancer Res. 2018;7(3):S254‐s257. 30393616 10.21037/tlcr.2018.09.06 PMC6193924

[249] Weerasinghe P , Garcia GE , Zhu Q , et al. Inhibition of Stat3 activation and tumor growth suppression of non‐small cell lung cancer by G‐quartet oligonucleotides. Int J Oncol. 2007;31(1):129‐136. 17549413

[250] Jing N , Zhu Q , Yuan P , Li Y , Mao L , Tweardy DJ . Targeting signal transducer and activator of transcription 3 with G‐quartet oligonucleotides: a potential novel therapy for head and neck cancer. Mol Cancer Ther. 2006;5(2):279‐286. 16505101 10.1158/1535-7163.MCT-05-0302

[251] Huang W , Dong Z , Chen Y , et al. Small‐molecule inhibitors targeting the DNA‐binding domain of STAT3 suppress tumor growth, metastasis and STAT3 target gene expression in vivo. Oncogene. 2016;35(6):783‐792. 26073084 10.1038/onc.2015.215

[252] Huang W , Dong Z , Wang F , Peng H , Liu J‐Y , Zhang J‐T . A small molecule compound targeting STAT3 DNA‐binding domain inhibits cancer cell proliferation, migration, and invasion. ACS Chem Biol. 2014;9(5):1188‐1196. 24661007 10.1021/cb500071v PMC4033648

[253] Jonker DJ , Nott L , Yoshino T , et al. Napabucasin versus placebo in refractory advanced colorectal cancer: a randomised phase 3 trial. Lancet Gastroenterol Hepatol. 2018;3(4):263‐270. 29397354 10.1016/S2468-1253(18)30009-8

[254] Shitara K , Yodo Y , Iino S . A phase I study of napabucasin plus paclitaxel for Japanese patients with advanced/recurrent gastric cancer. In Vivo. 2019;33(3):933‐937. 31028219 10.21873/invivo.11561 PMC6559899

[255] Han D , Yu T , Dong N , Wang B , Sun F , Jiang D . Napabucasin, a novel STAT3 inhibitor suppresses proliferation, invasion and stemness of glioblastoma cells. J Exp Clin Cancer Res. 2019;38(1):289. 31277685 10.1186/s13046-019-1289-6 PMC6612138

[256] Son DJ , Zheng J , Jung YY , et al. MMPP attenuates non‐small cell lung cancer growth by inhibiting the STAT3 DNA‐Binding activity via direct binding to the STAT3 DNA‐binding domain. Theranostics. 2017;7(18):4632‐4642. 29158850 10.7150/thno.18630 PMC5695154

[257] Zheng J , Son DJ , Lee HL , et al. E)‐2‐methoxy‐4‐(3‐(4‐methoxyphenyl)prop‐1‐en‐1‐yl)phenol suppresses ovarian cancer cell growth via inhibition of ERK and STAT3. Mol Carcinog. 2017;56(9):2003‐2013. 28277616 10.1002/mc.22648

[258] Hong D , Kurzrock R , Kim Y , et al. AZD9150, a next‐generation antisense oligonucleotide inhibitor of STAT3 with early evidence of clinical activity in lymphoma and lung cancer. Sci Transl Med. 2015;7(314):314ra185. 10.1126/scitranslmed.aac5272 PMC5279222 26582900

[259] Sun CY , Nie J , Huang JP , Zheng GJ , Feng B . Targeting STAT3 inhibition to reverse cisplatin resistance. Biomed Pharmacother. 2019;117:109135. 31226634 10.1016/j.biopha.2019.109135

[260] Xu S , Zhao N , Hui L , Song M , Miao ZW , Jiang XJ . MicroRNA‐124‐3p inhibits the growth and metastasis of nasopharyngeal carcinoma cells by targeting STAT3. Oncol Rep. 2016;35(3):1385‐1394. 26707908 10.3892/or.2015.4524

[261] Bai L , Zhou H , Xu R , et al. A potent and selective small‐molecule degrader of STAT3 achieves complete tumor regression in vivo. Cancer Cell. 2019;36(5):498‐511 e17. 10.1016/j.ccell.2019.10.002 PMC6880868 31715132

[262] Gaykalova DA , Manola JB , Ozawa H , et al. NF‐kappaB and stat3 transcription factor signatures differentiate HPV‐positive and HPV‐negative head and neck squamous cell carcinoma. Int J Cancer. 2015;137(8):1879‐1889. 25857630 10.1002/ijc.29558 PMC4629062

[263] Cordes F , Foell D , Ding JN , Varga G , Bettenworth D . Differential regulation of JAK/STAT‐signaling in patients with ulcerative colitis and Crohn's disease. World J Gastroenterol. 2020;26(28):4055‐4075. 32821070 10.3748/wjg.v26.i28.4055 PMC7403801

[264] Luo W , Li YX , Jiang LJ , Chen Q , Wang T , Ye DW . Targeting JAK‐STAT signaling to control cytokine release syndrome in COVID‐19. Trends Pharmacol Sci. 2020;41(8):531‐543. 32580895 10.1016/j.tips.2020.06.007 PMC7298494

[265] Huynh J , Etemadi N , Hollande F , Ernst M , Buchert M . The JAK/STAT3 axis: a comprehensive drug target for solid malignancies. Semin Cancer Biol. 2017;45:13‐22. 10.1016/j.semcancer.2017.06.001 28647610

[266] Pinto N , Prokopec SD , Vizeacoumar F , et al. Lestaurtinib is a potent inhibitor of anaplastic thyroid cancer cell line models. PLoS One. 2018;13(11):e0207152. 30419054 10.1371/journal.pone.0207152 PMC6231667

[267] Minturn JE , Evans AE , Villablanca JG , et al. Phase I trial of lestaurtinib for children with refractory neuroblastoma: a new approaches to neuroblastoma therapy consortium study. Cancer Chemother Pharmacol. 2011;68(4):1057‐1065. 21340605 10.1007/s00280-011-1581-4 PMC4238911

[268] Knapper S , Burnett AK , Littlewood T , et al. A phase 2 trial of the FLT3 inhibitor lestaurtinib (CEP701) as first‐line treatment for older patients with acute myeloid leukemia not considered fit for intensive chemotherapy. Blood. 2006;108(10):3262‐3270. 16857985 10.1182/blood-2006-04-015560

[269] Knapper S , Russell N , Gilkes A , et al. A randomized assessment of adding the kinase inhibitor lestaurtinib to first‐line chemotherapy for FLT3‐mutated AML. Blood. 2017;129(9):1143‐1154. 27872058 10.1182/blood-2016-07-730648 PMC5364440

[270] Plimack ER , Lorusso PM , McCoon P , et al. AZD1480: a phase I study of a novel JAK2 inhibitor in solid tumors. Oncologist. 2013;18(7):819‐820. 23847256 10.1634/theoncologist.2013-0198 PMC3720635

[271] Tsujita Y , Horiguchi A , Tasaki S , et al. STAT3 inhibition by WP1066 suppresses the growth and invasiveness of bladder cancer cells. Oncol Rep. 2017;38(4):2197‐2204. 28849140 10.3892/or.2017.5902

[272] Ferrajoli A , Faderl S , Van Q , et al. WP1066 disrupts Janus kinase‐2 and induces caspase‐dependent apoptosis in acute myelogenous leukemia cells. Cancer Res. 2007;67(23):11291‐11299. 18056455 10.1158/0008-5472.CAN-07-0593

[273] Sau S , Mondal SK , Kashaw SK , Iyer AK , Banerjee R . Combination of cationic dexamethasone derivative and STAT3 inhibitor (WP1066) for aggressive melanoma: a strategy for repurposing a phase I clinical trial drug. Mol Cell Biochem. 2017;436(1):119‐136. 28585089 10.1007/s11010-017-3084-z

[274] Liu JF , Deng WW , Chen L , et al. Inhibition of JAK2/STAT3 reduces tumor‐induced angiogenesis and myeloid‐derived suppressor cells in head and neck cancer. Mol Carcinog. 2018;57(3):429‐439. 29215754 10.1002/mc.22767

[275] Dao KT , Gotlib J , Deininger MMN , et al. Efficacy of ruxolitinib in patients with chronic neutrophilic leukemia and atypical chronic myeloid leukemia. J Clin Oncol. 2020;38(10):1006‐1018. 31880950 10.1200/JCO.19.00895 PMC7106977

[276] Mohan CD , Rangappa S , Preetham HD , et al. Targeting STAT3 signaling pathway in cancer by agents derived from Mother Nature. Semin Cancer Biol. Published online April 20, 2020. doi: 10.1016/j.semcancer.2020.03.016 10.1016/j.semcancer.2020.03.016 32325172

[277] Chen S , Bai Y , Li Z , et al. A betulinic acid derivative SH479 inhibits collagen‐induced arthritis by modulating T cell differentiation and cytokine balance. Biochem Pharmacol. 2017;126:69‐78. 27965071 10.1016/j.bcp.2016.12.006

[278] Lee JH , Kim C , Lee J , Um JY , Sethi G , Ahn KS . Arctiin is a pharmacological inhibitor of STAT3 phosphorylation at tyrosine 705 residue and potentiates bortezomib‐induced apoptotic and anti‐angiogenic effects in human multiple myeloma cells. Phytomedicine. 2019;55:282‐292. 30668440 10.1016/j.phymed.2018.06.038

[279] Rincon M . Interleukin‐6: from an inflammatory marker to a target for inflammatory diseases. Trends Immunol. 2012;33(11):571‐577. 22883707 10.1016/j.it.2012.07.003

[280] Stanam A , Love‐Homan L , Joseph TS , Espinosa‐Cotton M , Simons AL . Upregulated interleukin‐6 expression contributes to erlotinib resistance in head and neck squamous cell carcinoma. Mol Oncol. 2015;9(7):1371‐1383. 25888065 10.1016/j.molonc.2015.03.008 PMC4523436

[281] Nguyen MLT , Bui KC , Scholta T , et al. Targeting interleukin 6 signaling by monoclonal antibody siltuximab on cholangiocarcinoma. J Gastroenterol Hepatol. 2021;36(5):1334‐1345. 33091158 10.1111/jgh.15307

[282] Song L , Smith MA , Doshi P , et al. Antitumor efficacy of the anti‐interleukin‐6 (IL‐6) antibody siltuximab in mouse xenograft models of lung cancer. J Thorac Oncol. 2014;9(7):974‐982. 24922005 10.1097/JTO.0000000000000193 PMC4057975

[283] Angevin E , Tabernero J , Elez E , et al. A phase I/II, multiple‐dose, dose‐escalation study of siltuximab, an anti‐interleukin‐6 monoclonal antibody, in patients with advanced solid tumors. Clin Cancer Res. 2014;20(8):2192‐2204. 24563479 10.1158/1078-0432.CCR-13-2200

[284] Coward J , Kulbe H , Chakravarty P , et al. Interleukin‐6 as a therapeutic target in human ovarian cancer. Clin Cancer Res. 2011;17(18):6083‐6096. 21795409 10.1158/1078-0432.CCR-11-0945 PMC3182554

[285] Fizazi K , De Bono JS , Flechon A , et al. Randomised phase II study of siltuximab (CNTO 328), an anti‐IL‐6 monoclonal antibody, in combination with mitoxantrone/prednisone versus mitoxantrone/prednisone alone in metastatic castration‐resistant prostate cancer. Eur J Cancer. 2012;48(1):85‐93. 22129890 10.1016/j.ejca.2011.10.014

[286] Kraskouskaya D , Duodu E , Arpin CC , Gunning PT . Progress towards the development of SH2 domain inhibitors. Chem Soc Rev. 2013;42(8):3337‐3370. 23396540 10.1039/c3cs35449k

[287] Morlacchi P , Robertson FM , Klostergaard J , McMurray JS . Targeting SH2 domains in breast cancer. Future Med Chem. 2014;6(17):1909‐1926. 25495984 10.4155/fmc.14.120 PMC4339284

[288] Turkson J , Kim JS , Zhang S , et al. Novel peptidomimetic inhibitors of signal transducer and activator of transcription 3 dimerization and biological activity. Mol Cancer Ther. 2004;3(3):261‐269. 15026546

[289] Mandal PK , Limbrick D , Coleman DR , et al. Conformationally constrained peptidomimetic inhibitors of signal transducer and activator of transcription. 3: evaluation and molecular modeling. J Med Chem. 2009;52(8):2429‐2442. 19334714 10.1021/jm801491w PMC2735258

[290] McMurray JS , Mandal PK , Liao WS , Ren Z , Chen X . Inhibition of Stat3 by cell‐permeable peptidomimetic prodrugs targeted to its SH2 domain. Adv Exp Med Biol. 2009;611:545‐546. 19400306 10.1007/978-0-387-73657-0_239

[291] Qiu HY , Zhu X , Luo YL , et al. Identification of new shikonin derivatives as antitumor agents targeting STAT3 SH2 domain. Sci Rep. 2017;7(1):2863. 28588262 10.1038/s41598-017-02671-7 PMC5460289

[292] Kim BH , Lee H , Song Y , et al. Development of oxadiazole‐based ODZ10117 as a small‐molecule inhibitor of STAT3 for targeted cancer therapy. J Clin Med. 2019;8(11):1847. 10.3390/jcm8111847 PMC6912340 31684051

[293] Chen CL , Cen L , Kohout J , et al. Signal transducer and activator of transcription 3 activation is associated with bladder cancer cell growth and survival. Mol Cancer. 2008;7:78. 18939995 10.1186/1476-4598-7-78 PMC2577686

[294] Miyoshi K , Takaishi M , Nakajima K , et al. Stat3 as a therapeutic target for the treatment of psoriasis: a clinical feasibility study with STA‐21, a Stat3 inhibitor. J Invest Dermatol. 2011;131(1):108‐117. 20811392 10.1038/jid.2010.255

[295] Chen H , Bian A , Yang LF , et al. Targeting STAT3 by a small molecule suppresses pancreatic cancer progression. Oncogene. 2021;40(8):1440‐1457. 33420372 10.1038/s41388-020-01626-z PMC7906907

[296] Wang Y , Wang S , Wu Y , et al. Suppression of the growth and invasion of human head and neck squamous cell carcinomas via regulating STAT3 signaling and the miR‐21/beta‐catenin axis with HJC0152. Mol Cancer Ther. 2017;16(4):578‐590. 28138036 10.1158/1535-7163.MCT-16-0606 PMC5380531

[297] Yu PY , Gardner HL , Roberts R , et al. Target specificity, in vivo pharmacokinetics, and efficacy of the putative STAT3 inhibitor LY5 in osteosarcoma, Ewing's sarcoma, and rhabdomyosarcoma. PLoS One. 2017;12(7):e0181885. 28750090 10.1371/journal.pone.0181885 PMC5531494

[298] Bu LL , Zhao ZL , Liu JF , et al. STAT3 blockade enhances the efficacy of conventional chemotherapeutic agents by eradicating head neck stemloid cancer cell. Oncotarget. 2015;6(39):41944‐41958. 26556875 10.18632/oncotarget.5986 PMC4747200

[299] Furtek SL , Backos DS , Matheson CJ , Reigan P . Strategies and approaches of targeting STAT3 for cancer treatment. ACS Chem Biol. 2016;11(2):308‐318. 26730496 10.1021/acschembio.5b00945

[300] Leung CH , Chan DS , Ma VP , Ma DL . DNA‐binding small molecules as inhibitors of transcription factors. Med Res Rev. 2013;33(4):823‐846. 22549740 10.1002/med.21266

[301] Turkson J , Zhang S , Palmer J , et al. Inhibition of constitutive signal transducer and activator of transcription 3 activation by novel platinum complexes with potent antitumor activity. Mol Cancer Ther. 2004;3(12):1533‐1542. 15634646

[302] Cho SH , Park MH , Lee HP , et al. E)‐2,4‐Bis(p‐hydroxyphenyl)‐2‐butenal enhanced TRAIL‐induced apoptosis in ovarian cancer cells through downregulation of NF‐κB/STAT3 pathway. Arch Pharm Res. 2014;37(5):652‐661. 24390815 10.1007/s12272-013-0326-9

[303] Xi S , Gooding WE , Grandis JR . In vivo antitumor efficacy of STAT3 blockade using a transcription factor decoy approach: implications for cancer therapy. Oncogene. 2005;24(6):970‐979. 15592503 10.1038/sj.onc.1208316

[304] Lau YK , Ramaiyer M , Johnson DE , Grandis JR . Targeting STAT3 in cancer with nucleotide therapeutics. Cancers (Basel). 2019;11(11). 10.3390/cancers11111681 PMC6896109 31671769

[305] Shiah JV , Grandis JR , Johnson DE . Targeting STAT3 with proteolysis targeting chimeras and next‐generation antisense oligonucleotides. Mol Cancer Ther. 2021;20(2):219‐228. 33203730 10.1158/1535-7163.MCT-20-0599 PMC7888537

[306] Li WC , Ye SL , Sun RX , et al. Inhibition of growth and metastasis of human hepatocellular carcinoma by antisense oligonucleotide targeting signal transducer and activator of transcription 3. Clin Cancer Res. 2006;12(23):7140‐7148. 17145839 10.1158/1078-0432.CCR-06-0484

[307] Oweida AJ , Darragh L , Phan A , et al. STAT3 modulation of regulatory T cells in response to radiation therapy in head and neck cancer. J Natl Cancer Inst. 2019;111(12):1339‐1349. 30863843 10.1093/jnci/djz036 PMC6910208

[308] Brown PA , Kairalla JA , Hilden JM , et al. FLT3 inhibitor lestaurtinib plus chemotherapy for newly diagnosed KMT2A‐rearranged infant acute lymphoblastic leukemia: Children's Oncology Group trial AALL0631. Leukemia. 2021;35(5):1279‐1290. 33623141 10.1038/s41375-021-01177-6 PMC8763141

[309] Chan E , Mulkerin D , Rothenberg M , et al. A phase I trial of CEP‐701 + gemcitabine in patients with advanced adenocarcinoma of the pancreas. Invest New Drugs. 2008;26(3):241‐247. 18217204 10.1007/s10637-008-9118-3

[310] Argiris A , Duffy AG , Kummar S , et al. Early tumor progression associated with enhanced EGFR signaling with bortezomib, cetuximab, and radiotherapy for head and neck cancer. Clin Cancer Res. 2011;17(17):5755‐5764. 21750205 10.1158/1078-0432.CCR-11-0861 PMC3368806

[311] Harari PM , Harris J , Kies MS , et al. Postoperative chemoradiotherapy and cetuximab for high‐risk squamous cell carcinoma of the head and neck: Radiation Therapy Oncology Group RTOG‐0234. J Clin Oncol. 2014;32(23):2486‐2495. 25002723 10.1200/JCO.2013.53.9163 PMC4121506

[312] Sen M , Thomas SM , Kim S , et al. First‐in‐human trial of a STAT3 decoy oligonucleotide in head and neck tumors: implications for cancer therapy. Cancer Discov. 2012;2(8):694‐705. 22719020 10.1158/2159-8290.CD-12-0191 PMC3668699

[313] Reilley MJ , McCoon P , Cook C , et al. STAT3 antisense oligonucleotide AZD9150 in a subset of patients with heavily pretreated lymphoma: results of a phase 1b trial. J Immunother Cancer. 2018;6(1):119. 30446007 10.1186/s40425-018-0436-5 PMC6240242

[314] Bharadwaj U , Kasembeli MM , Robinson P , Tweardy DJ . Targeting Janus kinases and signal transducer and activator of transcription 3 to treat inflammation, fibrosis, and cancer: rationale, progress, and caution. Pharmacol Rev. 2020;72(2):486‐526. 32198236 10.1124/pr.119.018440 PMC7300325

[315] Bosch‐Barrera J , Queralt B , Menendez JA . Targeting STAT3 with silibinin to improve cancer therapeutics. Cancer Treat Rev. 2017;58:61‐69. 28686955 10.1016/j.ctrv.2017.06.003

[316] Hui Y , Yi X , Hou F , et al. Role of nanoparticle mechanical properties in cancer drug delivery. ACS Nano. 2019;13(7):7410‐7424. 31287659 10.1021/acsnano.9b03924

[317] Liu J , Zhang R , Xu ZP . Nanoparticle‐based nanomedicines to promote cancer immunotherapy: recent advances and future directions. Small. 2019;15(32):1900262. 10.1002/smll.201900262 30908864

[318] Jose A , Labala S , Ninave KM , Gade SK , Venuganti VVK . Effective skin cancer treatment by topical co‐delivery of curcumin and STAT3 siRNA using cationic liposomes. AAPS PharmSciTech. 2018;19(1):166‐175. 28639178 10.1208/s12249-017-0833-y

[319] Sun Y , Li X , Zhang L , et al. Cell permeable NBD peptide‐modified liposomes by hyaluronic acid coating for the synergistic targeted therapy of metastatic inflammatory breast cancer. Mol Pharmaceutics. 2019;16(3):1140‐1155. 10.1021/acs.molpharmaceut.8b01123 30668131

[320] Zhang J , Zhu S , Tan Q , et al. Combination therapy with ropivacaine‐loaded liposomes and nutrient deprivation for simultaneous cancer therapy and cancer pain relief. Theranostics. 2020;10(11):4885‐4899. 32308756 10.7150/thno.43932 PMC7163441

[321] Mohammadian J , Mahmoudi S , Pourmohammad P , et al. Formulation of Stattic as STAT3 inhibitor in nanostructured lipid carriers (NLCs) enhances efficacy of doxorubicin in melanoma cancer cells. Naunyn‐Schmiedeberg's Arch Pharmacol. 2020;393(12):2315‐2323. 32653978 10.1007/s00210-020-01942-x

[322] Bu L‐L , Wang H‐Q , Pan Y , et al. Gelatinase‐sensitive nanoparticles loaded with photosensitizer and STAT3 inhibitor for cancer photothermal therapy and immunotherapy. J Nanobiotechnology. 2021;19(1):1‐13. 34802438 10.1186/s12951-021-01125-7 PMC8607679

[323] Sivák L , Šubr V , Kovářová J , et al. Polymer‐ritonavir derivate nanomedicine with pH‐sensitive activation possesses potent anti‐tumor activity in vivo via inhibition of proteasome and STAT3 signaling. J Controlled Release. 2021;332:563‐580. 10.1016/j.jconrel.2021.03.015 33722611

[324] Tavares MR , Hrabánková K , Konefał R , et al. HPMA‐based copolymers carrying STAT3 inhibitor cucurbitacin‐D as stimulus‐sensitive nanomedicines for oncotherapy. Pharmaceutics. 2021;13(2):179. 33525658 10.3390/pharmaceutics13020179 PMC7911143

[325] Luo K , Gao Y , Yin S , et al. Co‐delivery of paclitaxel and STAT3 siRNA by a multifunctional nanocomplex for targeted treatment of metastatic breast cancer. Acta Biomater. 2021;134:649‐663. 34289420 10.1016/j.actbio.2021.07.029

[326] Zhuang X , Xiang X , Grizzle W , et al. Treatment of brain inflammatory diseases by delivering exosome encapsulated anti‐inflammatory drugs from the nasal region to the brain. Mol Ther. 2011;19(10):1769‐1779. 21915101 10.1038/mt.2011.164 PMC3188748

[327] Hosseini M , Baghaei K , Amani D , Ebtekar M . Tumor‐derived exosomes encapsulating miR‐34a promote apoptosis and inhibit migration and tumor progression of colorectal cancer cells under in vitro condition. DARU J Pharm Sci. 2021;29(2):267‐278. 10.1007/s40199-021-00400-0 PMC8602593 34405380

[328] Andersen MN , Etzerodt A , Graversen JH , et al. STAT3 inhibition specifically in human monocytes and macrophages by CD163‐targeted corosolic acid‐containing liposomes. Cancer Immunol Immunother. 2019;68(3):489‐502. 30637473 10.1007/s00262-019-02301-3 PMC11028169

[329] Guo C , Chen Y , Gao W , et al. Liposomal nanoparticles carrying anti‐IL6R antibody to the tumour microenvironment inhibit metastasis in two molecular subtypes of breast cancer mouse models. Theranostics. 2017;7(3):775. 28255366 10.7150/thno.17237 PMC5327649

[330] Shi K , Xue J , Fang Y , et al. Inorganic kernel‐reconstituted lipoprotein biomimetic nanovehicles enable efficient targeting “Trojan horse” delivery of STAT3‐Decoy oligonucleotide for overcoming TRAIL resistance. Theranostics. 2017;7(18):4480. 29158840 10.7150/thno.21707 PMC5695144

[331] Zhang S , Gupta S , Fitzgerald TJ . Dual radiosensitization and anti‐STAT3 anti‐proliferative strategy based on delivery of gold nanoparticle ‐ oligonucleotide nanoconstructs to head and neck cancer cells. Nanotheranostics. 2018;2(1):1‐11. 29291159 10.7150/ntno.22335 PMC5743834

[332] Ngamcherdtrakul W , Reda M , Nelson MA , et al. In situ tumor vaccination with nanoparticle co‐delivering CpG and STAT3 siRNA to effectively induce whole‐body antitumor immune response. Adv Mater. 2021:2100628. 10.1002/adma.202100628 PMC8424660 34118167

[333] Labala S , Jose A , Chawla SR , et al. Effective melanoma cancer suppression by iontophoretic co‐delivery of STAT3 siRNA and imatinib using gold nanoparticles. Int J Pharm. 2017;525(2):407‐417. 28373100 10.1016/j.ijpharm.2017.03.087

[334] Budi HS , Izadi S , Timoshin A , et al. Blockade of HIF‐1α and STAT3 by hyaluronate‐conjugated TAT‐chitosan‐SPION nanoparticles loaded with siRNA molecules prevents tumor growth. Nanomed Nanotechnol Biol Med. 2021;34:102373. 10.1016/j.nano.2021.102373 33667724

[335] Malam Y , Loizidou M , Seifalian AM . Liposomes and nanoparticles: nanosized vehicles for drug delivery in cancer. Trends Pharmacol Sci. 2009;30(11):592‐599. 19837467 10.1016/j.tips.2009.08.004

[336] Wu X , Tang W , Marquez RT , et al. Overcoming chemo/radio‐resistance of pancreatic cancer by inhibiting STAT3 signaling. Oncotarget. 2016;7(10):11708‐11723. 26887043 10.18632/oncotarget.7336 PMC4905505

[337] Zheng H , Chen Z , Cai A , et al. Nanoparticle mediated codelivery of nifuratel and doxorubicin for synergistic anticancer therapy through STAT3 inhibition. Colloids Surf B Biointerfaces. 2020;193:111109. 32416521 10.1016/j.colsurfb.2020.111109

[338] Garg SM , Vakili MR , Molavi O , Lavasanifar A . Self‐associating poly(ethylene oxide)‐block‐poly(alpha‐carboxyl‐epsilon‐caprolactone) drug conjugates for the delivery of STAT3 inhibitor JSI‐124: potential application in cancer immunotherapy. Mol Pharm. 2017;14(8):2570‐2584. 28221800 10.1021/acs.molpharmaceut.6b01119

[339] Molavi O , Mahmud A , Hamdy S , et al. Development of a poly(d,l‐lactic‐co‐glycolic acid) nanoparticle formulation of STAT3 inhibitor JSI‐124: implication for cancer immunotherapy. Mol Pharm. 2010;7(2):364‐374. 20030320 10.1021/mp900145g

[340] Molavi O , Ma Z , Mahmud A , et al. Polymeric micelles for the solubilization and delivery of STAT3 inhibitor cucurbitacins in solid tumors. Int J Pharm. 2008;347(1‐2):118‐127. 17681440 10.1016/j.ijpharm.2007.06.032 PMC2663961

[341] Pore N , Wu S , Standifer N , et al. Resistance to durvalumab and durvalumab plus tremelimumab is associated with functional STK11 mutations in patients with non‐small cell lung cancer and is reversed by STAT3 knockdown. Cancer Discov. 2021;11(11):2828‐2845. 34230008 10.1158/2159-8290.CD-20-1543

[342] Lee DW , Gardner R , Porter DL , et al. Current concepts in the diagnosis and management of cytokine release syndrome. Blood. 2014;124(2):188‐195. 24876563 10.1182/blood-2014-05-552729 PMC4093680

[343] Guha P , Gardell J , Darpolor J , et al. STAT3 inhibition induces Bax‐dependent apoptosis in liver tumor myeloid‐derived suppressor cells. Oncogene. 2019;38(4):533‐548. 30158673 10.1038/s41388-018-0449-z

[344] Chan HCS , Shan H , Dahoun T , Vogel H , Yuan S . Advancing drug discovery via artificial intelligence. Trends Pharmacol Sci. 2019;40(8):592‐604. 31320117 10.1016/j.tips.2019.06.004

