# Erratum

**DOI:** 10.1002/mco2.135

**Published:** 2022-04-20

On first publication online of MCO2124^1^, the two labels of Figure 5 were written backwards and is now replaced with a new figure. In Introduction, “According to World Health Statistics 2021 posted by the World Health Organization, there were 1.9 million new cases diagnosed in 2021. Even in high‐income countries, cancer has been regarded as one of the leading causes of “premature death” defined as the death between the ages of 30 and 70.^2^” has been changed to “According to World Health Statistics 2021 posted by the World Health Organization, even in high‐income countries, cancer has been regarded as one of the leading causes of “premature death” defined as the death between the ages of 30 and 70.^2^”

**FIGURE 5:**
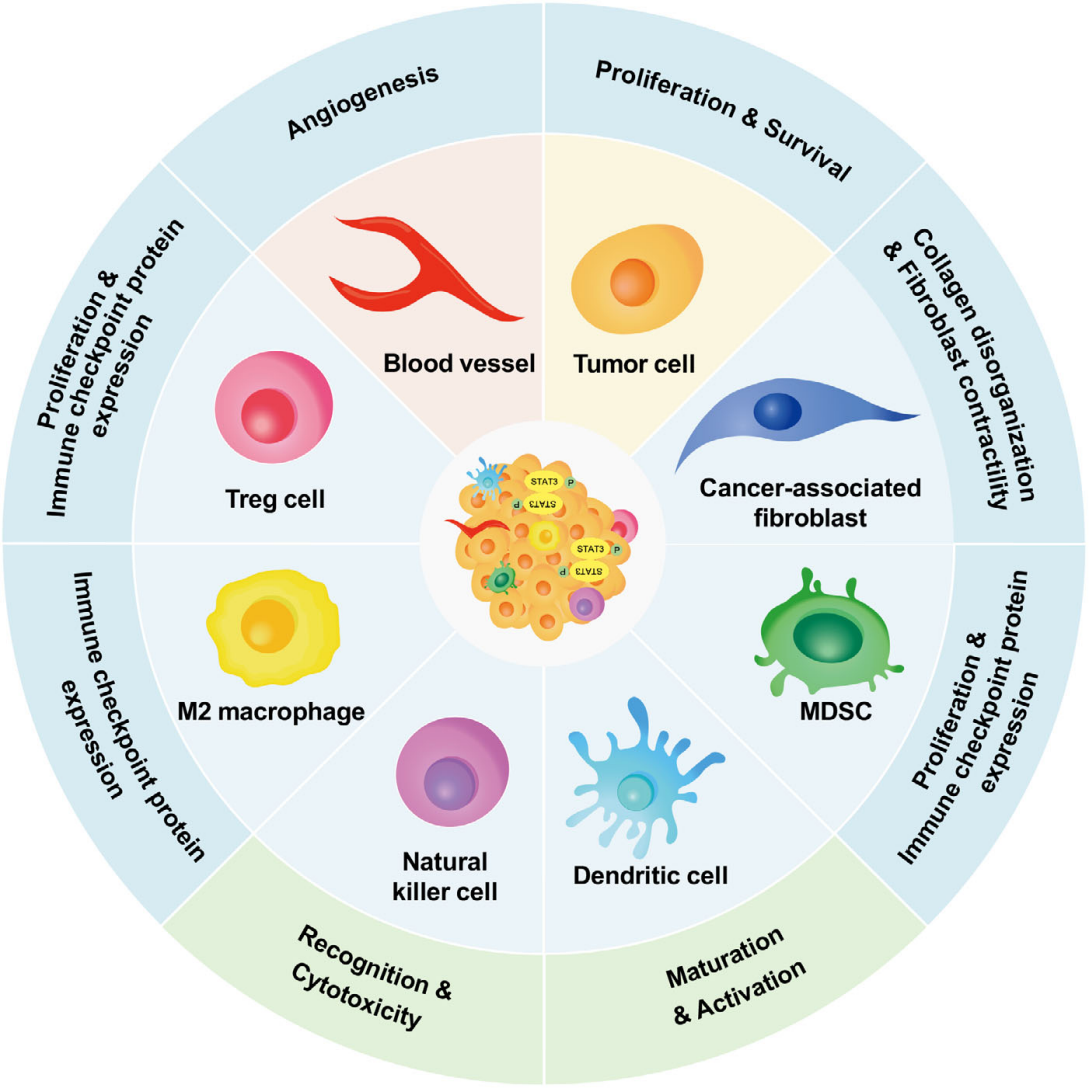
Schematic of STAT3 signaling in tumor microenvironment (TME). STAT3 activation has the ability in affecting TME via up‐ or downregulating downstream molecules and promoting tumor cell proliferation and survival, angiogenesis, immune evasion as the result. The functions of natural killer cell and dendritic cell in antigen presentation and target cell recognition are inhibited. While macrophage polarization toward M2‐like endotype and the immune checkpoint expression and proliferation of myeloid‐derived suppressor cell, cancer‐associated fibroblasts, and regulatory T‐cell are promoted by phosphorylated STAT3

We regret the error.
